# Recommendations for Combining Brain-Computer Interface, Motor Imagery, and Virtual Reality in Upper Limb Stroke Rehabilitation: Qualitative Participatory Design Study

**DOI:** 10.2196/71789

**Published:** 2025-10-15

**Authors:** Inês Oliveira, Miguel Russo, Ana Isabel Almeida, Athanasios Vourvopoulos, Carla Mendes Pereira

**Affiliations:** 1 Health School, Polytechnic University of Setúbal, Edifício ESCE/ESS, Setúbal, 2914-503, Portugal, 351 968471517; 2 Physiotherapy Department, Alcoitão Rehabilitation Medicine Center, Alcabideche, Portugal; 3 Institute for Systems and Robotics (ISR-Lisboa), Lisbon, Portugal; 4 Bioengineering Department, Higher Technical Institute, University of Lisbon, Lisbon, Portugal; 5 Comprehensive Health Research Centre, NOVA University Lisbon, Lisbon, Portugal

**Keywords:** neurological rehabilitation, cerebrovascular disorders, upper extremity, brain-computer interfaces, virtual reality, qualitative research, user-centered design

## Abstract

**Background:**

The high incidence and prevalence of upper limb impairment post stroke highlights the need for advancements in rehabilitation. Brain-computer interfaces (BCIs) represent a promising technology by directly training the central nervous system. The integration of motor imagery (MI) and motor observation through virtual reality (VR) using BCIs provides valuable opportunities for rehabilitation. However, the diversity in intervention designs demonstrates the lack of guiding recommendations integrating neurorehabilitation principles for BCIs.

**Objective:**

This study aims to develop recommendations for BCI interventions using task specificity and ecological validity through simulated VR tasks for upper limb stroke survivors by gathering tacit knowledge from neurorehabilitation experts, patients’ experiences, and engineers’ expertise to ensure a comprehensive approach.

**Methods:**

A multiperspective qualitative study was conducted through collaborative design workshops involving stroke survivors (n=17), neurorehabilitation experts (n=13), and biomedical engineers (n=3), totaling 33 participants. This innovative approach aimed to actively engage stakeholders in developing multifaceted solutions for complex health interventions.

**Results:**

Six themes emerged from the thematic analysis: (1) importance of patient-centered approach, (2) clinical evaluation and patient selection, (3) recommendations for task design, (4) guidelines for structuring BCI intervention, (5) key factors influencing motivation, and (6) technology features. From these themes, the following recommendations (R) are established: (R1) MI-based VR-BCI interventions must be conducted through a patient-centered approach, based on individualized preferences, needs, and goals of the user, by an interdisciplinary team; (R2) selection criteria must include upper limb impairment, cognitive and communication assessment, and clinical traits, such as MI capacity, neglect, and depression must be assessed since they might influence intervention outcomes; (R3) tasks to perform should preferably be based on daily living activities, including unilateral and bilateral tasks, and a variety of tasks must be available for selection to ensure meaningfulness for the user and suitability to clinical traits; (R4) intervention must be structured by different progressing levels starting with simple, gross movements and adding complexity through additional movement features, cognitive demand, or MI difficulty; (R5) optimal levels of motivation must be sustained through task variability, gamification elements, and task demand adequacy; and (R6) multisensorial potential of MI-based VR-BCI must be effectively harnessed through the adequate adjustment of visual, haptic, and proprioceptive feedback modalities to the patient.

**Conclusions:**

Current results contribute to establishing clear guidelines on patient selection, task design, intervention structuring, motivation factors, and tailoring of sensory feedback. This framework presents a foundation for optimal implementation of VR-BCI–based interventions that associate MI and motor observation, optimizing cortical activity during the intervention, patients’ engagement, and clinical outcomes. Future research should explore the application of these guidelines for validation and investigate BCIs’ efficacy according to different combinations of patients’ profiles, task characteristics, and technology features.

## Introduction

### Background

Stroke is among the most common causes of disability worldwide and is the chronic neurological disease with the greatest impact at this level [1,2]. Among the multiple consequences of stroke, upper limb (UL) impairments are one of the most prevalent, with around 70% of stroke survivors showing altered arm function, 40% maintaining persistent functional disability [3], and only 5%-20% showing complete dexterity 6 months after stroke [4]. Motor control impairments result in altered movement dynamics, characterized by muscle synergies that reduce optimal motor performance and induce movement compensations [5,6].

Upper limb rehabilitation (ULR) after stroke is recognized as a complex challenge due to different factors, namely the multifactorial nature of the condition (eg, spasticity, decreased muscle strength, changes in somatosensation and perception) [7-9] and the nonlinear progression of disability over time. In addition, the complexity underlying ULR is also compounded by the need to define multiple parameters, including the type of intervention, dosage (such as duration, difficulty, and intensity), application format, and the underlying factorial paradigm, which involves different degrees of parameter interaction [10].

Multiple types of motor rehabilitation interventions have been studied throughout the years with the goal of reducing motor impairments and improving functional activities, making use of learning- and use-dependent mechanisms [11] to promote motor learning. Considering UL interventions, traditional or modified constraint-induced movement therapy, mental practice, robot-assisted movement therapy, and neuromuscular electrical stimulation are currently among the most recommended strategies [11-13]. Neuroplasticity is the structural or functional changes (or both) within neurons affecting their connectivity with each other that occur during learning after injury, being a key element of motor rehabilitation and motor acquisition [14]. Motor rehabilitation allows patients to learn,optimize and adapt their motor, sensory, and cognitive functioning through dosed, repetitive, goal-oriented, progressive, task- and context-specific training [11]. The combination of traditional therapy methods with technologies that enable the use of enriched environments, sensory stimulation, and task-specific training is a neuroplasticity promoter, enhancing functional recovery for stroke survivors [15].

Brain-computer interfaces (BCIs) have been increasingly studied over the last 20 years as a way of enhancing ULR by being a facilitator of intensive and repetitive practice [16]. BCI is a technology that allows the acquisition, analysis, and decoding of brain activity, translating it into communication and control commands for an interface [17].

Measuring and analyzing brain activity data is often done using electroencephalography (EEG) due to its noninvasive, portable, low-cost nature and high temporal resolution [16]. EEG enables the detection of various brain activity patterns, such as event-related potentials (eg, P300), steady-state visual evoked potentials, and event-related desynchronization (ERD) and event-related synchronization (ERS) that are associated with motor-related tasks [18]. Common paradigms for intervention using BCI are motor imagery (MI) and motor attempt, also associated with motor observation [7]. Studies using MI tasks frequently rely on ERD and ERS as a reliable method for detecting motor-related brain activity [19]. MI-based BCI operation is then associated with the ERD phenomena and the modulation of sensorimotor rhythms, linked to imagined limb movements. By providing feedback based on the patient’s brain activity—that is, neurofeedback—BCIs close the motor-sensory feedback loop during MI training, allowing users to actively perceive and modulate their brain activity [20]. Repeated practice combined with real-time feedback promotes neuroplasticity and facilitates functional recovery [21]. Kruse et al [22] conducted a systematic review and meta-analysis showing evidence that the addition of MI-based BCI to conventional therapy may enhance the improvement of UL function and brain function recovery. Although meta-analysis on different design characteristics of BCI interventions favors motor intention over mental practice [17,23], MI offers a unique opportunity for stroke survivors who are unable to move their extremities by attempting to stimulate the brain regions responsible for movement [22,24].

MI, also referred to as mental practice and recently action imagery [25], is described as training movements or tasks in an imagined way and without producing movement, with the specific aim of improving performance [26]. Recently, Bach et al [27] proposed a conceptualization of MI, not as purely motoric and based on the execution of the movement, but in its anticipated perceptual effects, suggesting motor imagination (or “effect imagery” as proposed by the authors) is driven by the desired perceptual outcomes of actions rather than the movements themselves.

MI application is extensively recommended as an adjunct to conventional therapy [28-32]. Neuroimaging findings provided evidence that MI and physical practice are functionally equivalent, that is, they recruit overlapping brain regions within the brain motor networks underlying motor preparation and execution, including premotor, parietal, primary somatosensory, and motor cortices [33,34]. Both MI and planning and execution of movement involve the activation of the cerebellum, putamen, inferior frontal gyrus, and supplementary motor area [35]. Thus, the relevance of MI practice lies in reinforcing the motor schemas involved in actual movement through the mental visualization of motor actions [26]. MI can involve explicit or implicit tasks, where the first involves active and conscious imagination of a particular movement, and the second subconsciously induced processes for task-imagery problem solving, such as mental rotation of an image [31].

The use of neurofeedback associated with BCI seems to promote changes not only at the neurophysiological level but also at the clinical level [36]. This can be provided through different modalities, such as visual, auditory, and tactile feedback [37]. Virtual reality (VR) is one of the feedback possibilities to combine with BCI [7,36], which allows users to experience being represented by an avatar body rendered through a 3D environment and interact with it in a realistic way [38]. Multiple meta-analyses have described the positive effects of using VR in ULR, enhancing not only motor function but also activities of daily living (ADLs) [39,40], and practice guidelines recommend its usage to improve stroke rehabilitation outcomes [30]. Immersive VR, when used as a complement to conventional therapy, can provide a suitable environment for practicing the repetitive and specific tasks needed to improve motor skills after stroke [41].

### Synergistic Mechanisms of Brain-Computer Interfaces, Virtual Reality, and Motor Imagery

Starting from the premise that one of the main goals of neurorehabilitation is to promote neuroplasticity and restore function, MI, VR, and BCI form an interconnected triad that mutually reinforce one another, representing a powerful approach to achieve this objective. The effectiveness of this approach relies on several interdependent dimensions that are dynamically interconnected and mutually influential, such as: (1) the user’s ability to elicit and modulate neural oscillations associated with sensorimotor processes during MI [42]; (2) the system’s capacity to accurately detect, process, and classify these signals [19]; and (3) the user’s motivation, engagement, and adherence throughout the training process [43]. Each component of the intervention triad influences one or more of these dimensions, creating a reinforcing cycle that amplifies the overall effectiveness of MI-VR-BCI interventions.

MI-based BCIs typically have lower transfer rates compared to other BCI paradigms [44], and achieving effective control requires prolonged training protocols involving simultaneous learning by both the user and the system. While the user must draw on and further develop existing MI skills, the system simultaneously enhances classification accuracy through optimized algorithms and machine learning techniques [19]. However, a considerable proportion of users (approximately 15%-30%) [45] might face difficulties in effectively controlling BCI systems using MI alone, a phenomenon known as BCI inefficiency. Despite this, some authors argue that BCI inefficiency may not solely stem from biological limitations but may also be influenced by inadequate training protocols, likely due to MI being cognitively demanding, unfamiliar to many users, and often associated with fatigue or boredom during prolonged training sessions [46]. VR can counteract these challenges by increasing engagement and facilitating more vivid and effective MI. For example, immersive VR environments have been shown to elicit stronger ERD, facilitate sensorimotor rhythm modulation, and improve BCI performance [47,48]. A key mechanism underlying these effects may be embodiment—the sense of owning and controlling the avatar —which arises from the interplay between body ownership, agency, and self-location [49], with Forster et al [50] also considering the close intertwining with presence. In addition, VR supports action observation, potentially activating the mirror neuron system, both of which are known contributors to neuroplasticity [51]. Closing the loop by providing feedback during MI observation enhances neuroplasticity by engaging pre- and postsynaptic summation mechanisms, thereby promoting motor learning [21]. Furthermore, VR can enhance motor learning, improve body awareness, and elicit kinesthetic sensations even in the absence of physical movement [49,52]. Clinically, this is highly relevant for stroke survivors, who often struggle with body perception and a reduced sense of control over their affected limbs.

VR is also able to leverage certain principles of motor learning, like the specificity of the task, intensity, repetition, and relevance, necessary factors for the development and strengthening of synapses and, consequently, for motor recovery [53,54]. Another aspect that favors the association of MI, BCI, and VR is the multisensory stimulation potential associated with the latter. This combination has the potential to enhance hand function by delivering targeted sensory feedback across different systems, such as somatosensory, visual, and auditory pathways, thereby promoting neuroplasticity [55]. This feedback may help reduce the discrepancy between motor intention and MI commonly observed in BCI applications, potentially increasing the overall effectiveness of the MI-VR-BCI approach. Although it presents multiple theoretical benefits, this paradigm remains recent and innovative, raising important questions regarding its design and implementation.

BCI-based technologies have a broad range of applications and are increasingly being researched, but there remains a gap between the technology and the end user [56]. The development of BCI systems for rehabilitation must consider patients’ needs while encompassing clinical applicability and technical feasibility [57]. Research states that practitioners and researchers need to involve users in creating solutions that consider factors such as convenience, ease of use, privacy, security, safety and quality standards, prioritizing users, and ergonomics [58]. Given these considerations, it is necessary to establish recommendations for the use of MI-VR-BCI and adjust the technology to meet the real needs of users and clinical practice [59]. To address the gaps in existing evidence, this study aimed to explore the insights of (1) neurorehabilitation experts regarding the most effective factors and principles of neurorehabilitation and neurophysiology relevant to adapting for BCI intervention; (2) stroke survivors to inform recommendations based on their needs, preferences, and motivation; and (3) biomedical engineers on the technical knowledge of the technology.

Using a collaborative methodology, the study sought to develop realistic recommendations for designing more effective interventions for the ULR in stroke survivors using BCI-based interventions combined with VR and MI.

## Methods

### Study Design

**Figure 1.**
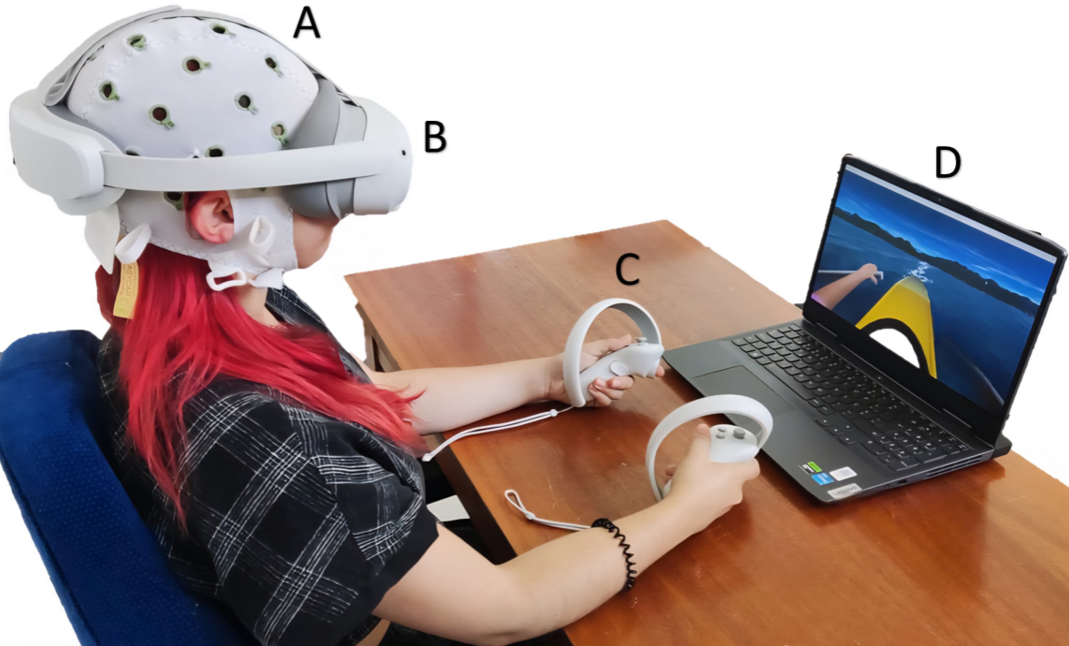
Set up for technology experimentation. (A) Electroencephalography cap. (B) Virtual reality headset (PICO 4). (C) Haptic feedback. (D) Scenario visualized through the virtual reality.

A qualitative design grounded in reflexive thematic analysis [60,61] was used within a constructionist epistemology and interpretivist theoretical perspective to draw from the tacit knowledge of experts and experience from stroke survivors through a reflexive thematic analysis. Thus, a multiperspective collaborative design process was used through workshops, including clinical neurorehabilitation experts, stroke survivors, and biomedical engineers [62]. For this study, both web-based and in-person workshops were planned. The first allowed the participation of geographically dispersed participants [63], and the second helped fully leverage interpersonal dynamics for enhanced collaboration and permitted experiencing the MI-VR paradigm. Technology experimentation was made through NeuRow developed by Vourvopoulos et al [64], a first-person perspective upper-limb MI training paradigm involving a bimanual boat-rowing task, with feedback delivered through a head-mounted-display VR and haptic feedback (Figure 1). Logistical and time constraints, particularly the extensive preparation and calibration required for the EEG, limited opportunities for technology experimentation including direct BCI control, only involving MI and VR. The COREQ (Consolidated Criteria for Reporting Qualitative Research) checklist was followed for this study (Multimedia Appendix 1) [65].

Workshop planning was based on the literature review conducted to familiarize with the topic and identify the evidence gaps. This search focused on meta-analyses in the population with stroke, including the MeSH (Medical Subject Headings) terms “stroke” AND “rehabilitation OR intervention OR therapy” and “upper extremity,” published in the last 5 years, and guidelines for rehabilitation and in meta-analysis on “Brain Computer Interface,” “Virtual Reality,” and “Motor Imagery.”

### Sample and Recruitment

The recruitment took place between January 2023 and July 2024, using a purposive sample. Participants were selected based on the researcher’s judgment [66], through the national stroke patient association’s email list, and the researchers’ professional networks. Considering the study goals, the priority in participant selection was the richness of contributions and good communication skills.

Neurorehabilitation experts were included if they had at least 10 years of experience with stroke rehabilitation. Stroke survivors were included if they (1) had a stroke-caused UL impairment, (2) were more than 18 years old at the time of the stroke and (3) had participated in at least 20 poststroke rehabilitation sessions with preferential previous technological experience either in therapy or daily life. Potential participants with stroke were excluded if they had a diagnosis of cognitive impairment or impaired communication (eg, aphasia) that limited their ability to understand complex instructions and to report their experience with stroke rehabilitation. The inclusion criteria for engineers were based on experience with BCI-based interventions and knowledge of the technical details.

Following an initial informal contact to gauge the willingness to participate, potential participants were sent an invitation letter, a consent form, a demographic characterization questionnaire, and scheduling options.

Of the 25 neurorehabilitation experts who were contacted, 21 expressed interest in participating; however, 8 were not able to participate due to scheduling difficulties. Among the 23 eligible stroke survivors interested in taking part, 6 could not attend due to scheduling issues. Of the 4 engineers who accepted, 1 was unable to attend due to illness. A total of 33 participants were recruited, including 13 neurorehabilitation experts, 17 stroke survivors, and 3 biomedical engineers, and sociodemographic information was collected (Tables1^3^; questionnaires for stroke survivor characterization in the Multimedia Appendix 1).

**Table 1.** Description of participants (neurorehabilitation experts, n=13).

Characteristics	Value
Sex, n	
Male	8
Field of rehabilitation, n	
Occupational therapist	3
Physicians	1
Physiotherapist	7
Speech and language therapist	1
Psychologist	1
Clinical Experience with stroke (years), mean (SD)	16.1 (6.55)
Previous experience with virtual reality, n	
None	1
Slight contact	8
Some experience	3
Frequent use	1
Familiarity with technology for rehabilitation (n)	
None	1
Slight contact	7
Some experience	2
Frequent use	3

**Table 2.** Description of participants (stroke survivors, n=17)

Characteristics	Value, n	Mean (SD)
Sex (female)	10	—^a^
Age (years)	—^a^	54.8 (8.32)
Time post stroke (years)	—^a^	5.2 (4.58)
Early chronic (6-12 months)	5	12 (1.64)
Mid chronic (>12-60 months)	5	34 (13.88)
Late chronic (>60 months)	7	132.0 (25.64)
Perceived dependency		
Completely independent	2	—^a^
Slightly dependent	5	—^a^
Moderately dependent	6	—^a^
Highly dependent	4	—^a^
Perceived movement capacity		
Movement moderately useful for ADLs^b^	3	—^a^
Some movement although not useful for ADLs	11	—^a^
Unable to move	3	—^a^
Previous virtual reality experience		
Yes, before the stroke	5	—^a^
Yes, after the stroke	3	—^a^
No	9	—^a^

a Not applicable.

b ADL: activity of daily living.

**Table 3.** Description of participants (biomedical engineers, n=3).

Characteristics	Value, n
Sex	
Female	2
Academic degree	
Doctorate	2
Bachelor	1
Experience with BCI^a^ systems development	
Some experience	1
Moderate	1
Moderate to high	1
Experience with technology for rehabilitation development	
Some experience	2
Moderate to high	1
Experience with VR^b^ in stroke rehabilitation	
None	1
Moderate	1
Moderate to high	1

a BCI: brain-computer interface.

b VR: virtual reality.

### Data Collection

A total of 6 workshops were conducted. Of them, 3 with neurorehabilitation experts (NEs), 2 with stroke survivors (SSs), and 1 with the participation of neurorehabilitation experts, stroke survivors, and biomedical engineers (BEs). 4 workshops were held remotely, and 2 workshops were conducted in person (workshops 5 and 6). Workshop durations ranged from 90 to 145 minutes. Each session followed a format with four key phases: (1) introduction outlining the workshop’s objectives, structure, and rules, relevant theoretical background, and summary of prior findings; (2) participant introductions and icebreaker activity; (3) core activities tailored to each workshop’s objectives; and (4) completion of a workshop standardized feedback questionnaire.

**Figure 2.**
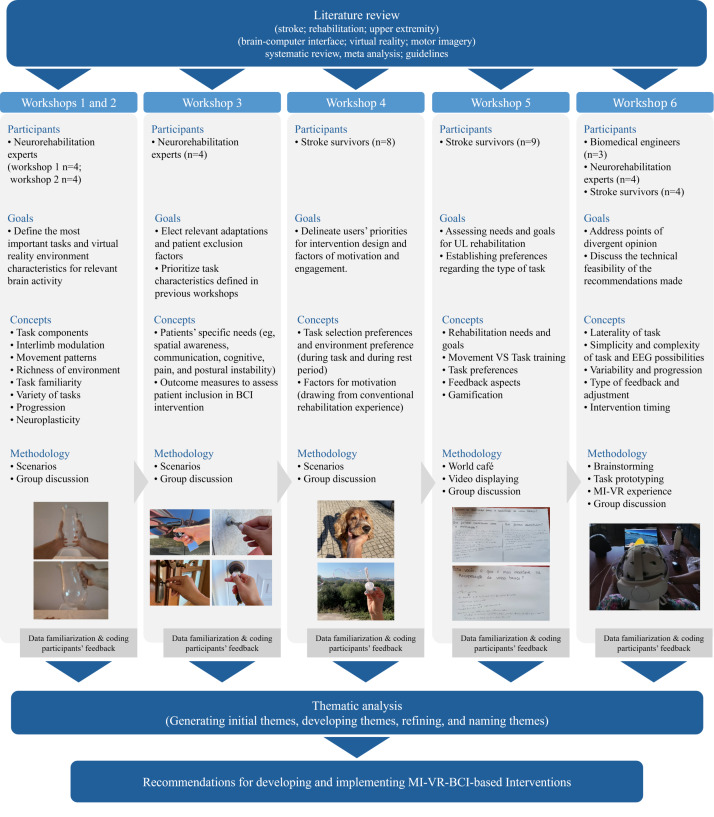
Workshop development and implementation process. The images represent methods and materials used during each workshop. BCI: brain–computer interface; EEG: electroencephalography; MI: motor imagery; UL: upper limb; VR: virtual reality.

For each workshop, specific goals were established, and corresponding methodologies were selected based on the recommendation of Ozkaynak et al [62] and Benson et al [67]. Accordingly, multisensory elicitation activities and complementary methods were used to enhance participant engagement, leverage diverse strengths, and maximize workshop productivity and efficacy. The methodologies used included scenarios, task prototyping, World Café, and technology experimentation, implemented either in plenary formats (workshops 1-4) or, where appropriate, also in small groups (workshops 5 and 6)—involving all different stakeholders in the case of the final workshop. The workshops were planned iteratively, with the outcomes of each session informing the next to address any gaps, clarify inconclusive points, or resolve disagreements. Figure 2 presents an overview of the workshops conducted, and a more detailed description is available at Table S1 (Multimedia Appendix 1).

To ensure consistency and comparability across sessions, the research team carefully predefined and pretested the structure and focus of each workshop while also ensuring researcher training. While tailored to each workshop’s specific goals and participant profiles, visual prompts and guiding questions were carefully designed and refined in advance by the research team. Summaries of each workshop were presented in the subsequent session to promote continuity. For workshops 5 and 6, a written summary was also shared with participants for validation. After each workshop, participants had the opportunity to provide feedback on their experience through a form with both closed- and open-ended questions and share their thoughts on the most important topics discussed, suggest any missing topics, and leave any other comments (Workshop Assessment Questionnaire in Multimedia Appendix 1). The analysis of the feedback questionnaires was used to plan the subsequent workshop.

### Data Analysis

An inductive thematic analysis was conducted using data from the verbatim transcripts [68]. The 6-phase process of thematic analysis was followed: data familiarization, systematic data coding, generating initial themes, developing themes, refining and naming themes, and producing the report [60].

The workshops were initially transcribed using the Microsoft Office Word transcribe feature, followed by manual review. Each transcript was scanned for relevant quotes, which were lifted into a codebook to group them into preliminary themes according to the study’s goals.

The preliminary themes were combined and reassembled into different themes and subthemes until the final thematic map was achieved. The analysis was validated by a second author and discussed with a third research member, followed by frequent debriefing sessions within the research team.

### Researcher Characteristics and Reflexivity

The research team was comprised of 4 physiotherapists and 1 biomedical engineer, with clinical experience and research experience in the field of stroke rehabilitation. They believe in stroke survivors’ potential to improve, the importance of their active role in their rehabilitation, and the importance of neurorehabilitation principles knowledge to support intervention. Although the research team considers the combination of MI-VR-BCI, a promising tool for upper limb stroke rehabilitation, they view it as a complement rather than a replacement for conventional rehabilitation, as supported by high-quality scientific literature. While this is the researchers’ perspective, a reflexive approach was used throughout the study’s conduct and data analysis, with continuous attention to positionality and potential bias. Data interpretation was conducted with transparency and critical awareness, and findings were presented in an open, reflective, and analytically rigorous manner.

Throughout the study, there was an introspective concern to maintain focus on the research question and relevant framework while promoting an environment of free participation by the participants. For the first 4 workshops, there was little to no prior contact and personal relationship with the participants. In the fifth and sixth workshops, a professional association existed between the researcher conducting the sessions and 6 patients, as well as 3 health care professionals, due to their shared involvement at the rehabilitation center. These pre-existing professional relationships did not influence the design, methodology, or conduct of the workshops.

To ensure the quality of the study, meetings were held for discussion of the methodology and analysis processes. This step reduced possible bias from the researchers and increased scientific rigor.

### Ethical Considerations

The study was approved by the Ethics Committee of the Polytechnic Institute of Setúbal (CE-IPS 35/2023) and Alcoitão Rehabilitation Medicine Center (CMRA2023/006 and CMRA 2023_012). All participants were informed of the aims of the study, procedure, risks, and confidentiality through an invitation letter. Prior to taking part in this study, the participants signed the informed consent form. Participants did not receive any form of financial or material compensation. Participants were also informed that workshops would be recorded, their identity would be kept confidential, and that they had the right to withdraw from this project at any time without any prejudice.

## Results

### Overview

**Figure 3.**
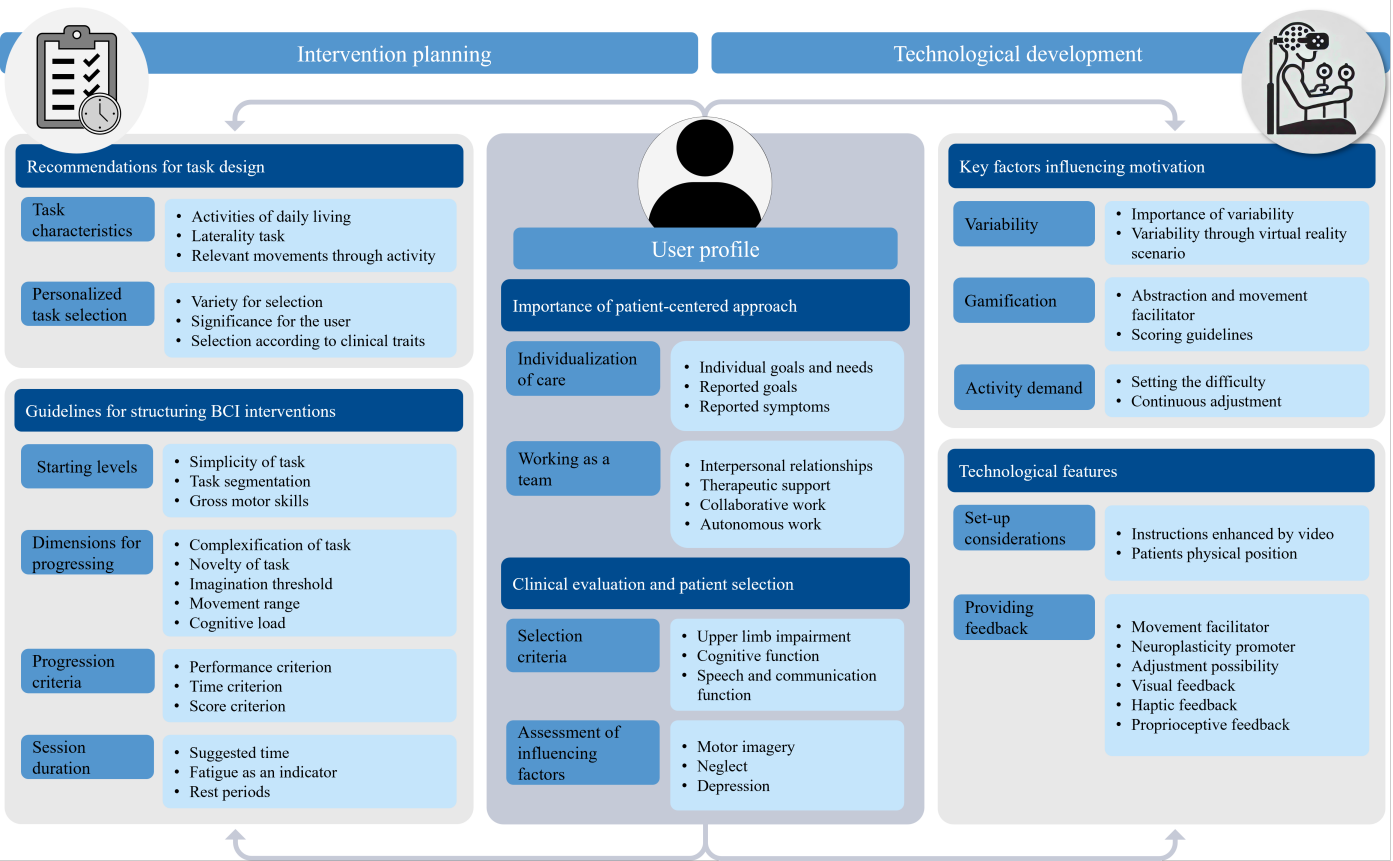
Illustration map of the themes, subthemes, codes, and how they interrelate. BCI: brain–computer interface.

The thematic analysis revealed 6 themes (Table S2 in Multimedia Appendix 1) regarding recommendations for planning and implementing MI-VR-BCI interventions and were arranged according to 3 dimensions: user profile (themes 1 and 2), intervention planning (themes 3 and 4), and technological development (themes 5 and 6), with all dimensions closely intertwined. The user profile dimension integrates the importance of a patient-centered approach and considerations on clinical evaluation and patient selection. The intervention planning dimension contemplates the recommendations for task design and guidelines for structuring BCI intervention. The technological development dimension considers the key factors influencing motivation and specific recommendations on technological features. A map was created to provide a comprehensive summary of the findings (Figure 3). Identification of the participant’s group (NE, SS, and BE) and number is used in quotations, followed by the identification of the workshop.

### Importance of Patient-Centered Approach

#### Individualization of Care

Participants emphasized that each stroke survivor presents unique, distinct needs and preferences, making it essential to center rehabilitation around the individual’s characteristics and goals. Key goals identified by stroke survivors included enhancing functionality (“the ability to perform daily tasks”) and achieving independence, or reducing dependency on others. Functionality was closely linked to autonomy, with the desire to perform daily tasks and social gestures, such as hugging, handshaking, or clapping being a significant motivator and critical target for their rehabilitation efforts.

Participants also emphasized the need to address structural impairments, such as manual dexterity, tactile sensory deficits, pain management, neglect, and perception. The diversity of symptoms and goals indicates that rehabilitation must be tailored to each stroke survivor’s priorities and challenges. This individualized approach should guide the design of tasks and technological features of MI-VR-BCI solutions, ensuring that different dimensions, including dexterity, perception, or motor planning, are addressed through targeted activities. By systematically assessing stroke survivors’ specific symptoms and goals, the rehabilitation process can be optimized for better outcomes and relevance.

*We have to put the patient here at the center. Not here near me... but in the center*.

[NE 13, workshop 6]

When asked to write down what is more important for arm recovery, stroke survivors wrote:

*Participate with agility in our grandchildren’s games.*

*Hugging and shaking hands.*

*Functional hand (for daily tasks like eating, dressing, and hygiene).*

*Regaining functional autonomy.*

[Group written conclusion, workshop 5]

#### Working as a Team

During the workshops, participants emphasized the importance of involving an interdisciplinary team in the intervention process. This collaboration enables specialists from different fields to support stroke survivors and guide decision-making, particularly during the assessment phase, to identify all possible influential factors and ensure optimal benefits. Stroke survivors should be active team members, participating equally in decisions. This collaborative approach enhances participant selection, clinical profile establishment, intervention outcomes, and the avoidance of adverse effects. Given the cutting-edge nature of BCI with extensive research exploration, participants also highlighted the importance of collaboration between clinical and research teams.

Regarding interpersonal relationships, stroke survivors valued being understood by health care professionals, which, along with positive feedback, enhanced their satisfaction and sense of contentment. Participants also stressed the importance of health care professionals being prepared to address the stroke survivor’s feelings of frustration and discomfort. Stroke survivors also highlighted the importance of therapeutic guidance and specialized attention, while also being empowered to work autonomously. Correspondingly, the end of sessions and associated time constraints were seen as demotivating, as they had to wait until the next session to continue their progress.

*It’s also important to have, realistically speaking, multidisciplinary teams so that we can all understand the patient.*

[NE 13, workshop 6]

When asked to write down what builds the sensation of a “Good Session,” stroke survivors wrote:

*Involvement between therapists and patients (ability for therapists to understand us).*

[Group written conclusion, workshop 5]

*I learn an exercise with the therapist [...] Then I want to train, I want to exercise.*

[SS 9, workshop 5]

### Clinical Evaluation and Patient Selection

#### Selection Criteria

When considering the inclusion in a clinical program or research setting, participants emphasized the importance of stroke survivors’ motivation to engage in MI-VR-BCI interventions and the need to establish the technology’s true potential benefits. Clarifying the meaningful therapeutic value and assessing various clinical aspects were noted as crucial.

For UL impairment assessment, the Fugl-Meyer Assessment of motor recovery after stroke was recommended. It was observed that stroke survivors with higher UL functioning levels might struggle with interventions involving only MI, which should be considered when determining the impairment level threshold for inclusion. The intrinsic connection between motor and cognitive function was also addressed, recommending a comprehensive cognitive assessment using the Montreal Cognitive Assessment (MoCA) to evaluate spatial, temporal, and personal orientation, as well as the ability to follow instructions. Stroke survivors lacking this ability should be excluded.

Language assessment, particularly comprehension, was emphasized due to its impact on stroke survivors’ ability to use the technology and understand tasks. Global aphasia was established as an exclusion criterion, with the ability to understand simple commands deemed essential. Given the nature of the intervention, which relies on mental practice rather than physical movement, participants stated that this approach offers inclusiveness, providing therapeutic options for stroke survivors often excluded from studies.

*If a person is disorientated in all three dimensions, they’re hardly going to be able to do anything. [RE 3: Exactly, exactly.] And we’re wasting our time in rehabilitation work […] we must understand what the person’s initial cognitive part is and then work on this motor part intensively.*

[NE 13, workshop 6]

*There’s so little [in studies] of this ability to include certain subgroups, that perhaps developing something for this fringe of users [... would be] quite relevant.*

[NE 1, workshop 1]

#### Assessment of Influencing Factors

Participants emphasized the importance of assessing imagery capacity, as it can directly influence intervention outcomes. While not considered an exclusion criterion, evaluating imagery capacity helps tailor the amount of imagery training required before starting the intervention. The assessment tools recommended by the participants include the Vividness of Visual Imagery Questionnaire, the Movement Imagery Questionnaire, and the Left-Right Judgment Task.

Affective changes and emotional stability may play a crucial role, as they impact motivation and intervention success. Participants recommended assessing depression and anxiety with tools like the Beck Depression Inventory or the Hospital Anxiety and Depression Scale. Emotional challenges, such as encountering activities that participants can no longer perform, may cause discomfort and need to be managed carefully.

Neglect was frequently highlighted as a clinical trait to consider in participant selection and intervention planning. Neglect can alter spatial and body perception, affecting interaction with the interface and performance of imagery tasks. While neglect may affect outcomes, participants emphasized that it should be assessed to adapt tasks rather than exclude participants. Concomitantly, many participants emphasized the potential of virtual reality as a neglect training tool, suggesting that appropriate adaptations should support participation.

*[...] The question of motivation: if the person is depressed, we can do all the work in the world, but if we don’t address the affective issue... the result may not be so positive. So we have to act on different fronts. It might be useful, or it should probably be compulsory to have a psychological assessment beforehand.*

[BE 1, workshop 6]

*[...] spatial organization, the ability to scan the image, the perception of space and body awareness... There are several things here that seem to be more cognitive than necessarily motor, because we’re considering just imagery.*

[NE 2, workshop 3]

### Recommendations for Task Design and Selection

#### Task Characteristics

Most stroke survivors and health care professionals preferred tasks related to ADLs due to their relevance and strong connection to “muscle memory,” which aids MI. These tasks were seen as familiar, mundane, or realistic, representing activities encountered in the real world and enhancing motivation in ULR sessions. A list of relevant activities may be found in Table S3 in Multimedia Appendix 1. Although for the majority, daily activity-based tasks were favored, some participants suggested that less common activities could also boost motivation and provide abstraction from the condition and UL movement difficulties.

Participants debated the relevance, advantages, and nuances of unilateral versus bilateral tasks, their influence on MI, motor planning, and neuroplasticity stimulation, with no clear consensus. Unilateral tasks were recommended for their potential to elicit specific brain activation and reduce interhemispheric inhibition—a phenomenon in which the healthy hemisphere exerts inhibitory influence on the hemisphere targeted for rehabilitation, possibly leading to counterproductive effects. Accordingly, bilateral activities would only start after mastery of unilateral ones (with the suggestion of starting with the less affected side). Conversely, some advocated for early integration of bilateral activities to support the learning in the lesioned side. Initiating tasks with the more affected side having a “supportive role” in the movement of the less affected one, according to an asymmetrical execution, is stated as being more similar to daily life tasks. Moreover, 1 participant stated that, in cases of severe dysfunction, symmetric tasks might be more adequate.

A combined approach of both unilateral and bilateral tasks within the same session was also suggested to leverage the benefits of each. It was emphasized that movements of the more affected side should occur more frequently, with randomized task order to prevent habituation. Respecting the natural execution of tasks (unilateral or bilateral) was deemed important to facilitate motor memory.

Specific activities to practice relevant movements were viewed as beneficial for movement planning and increased engagement. The movements and functions found relevant for stroke rehabilitation are listed on Table S4 in Multimedia Appendix 1. Tasks engaging the UL in a global and integrated manner were preferred, as they activate a wider range of brain circuits and align with everyday activities. Breaking down tasks into smaller segments was recommended to facilitate MI, especially at early stages.

*I always prefer training things that make sense to me in my day-to-day life, […] things that are useful*.

[SS 6, workshop 4]

*To reduce the competition in terms of the cerebral hemispheres I would say that it could be unilateral. However, we know that bilateral tasks sometimes favour active movement and rehabilitation. […] I would invite unilateral activity and quite possibly try to inhibit the contralesional side*.

[NE 2, workshop 1]

#### Personalized Task Selection

Participants defended a personalized approach to task selection, considering both user preferences and relevant clinical traits. They emphasized the importance of offering a variety of tasks to match the stroke survivor’s needs, goals, and preferences, resulting from a collaborative decision-making process between the health professional and the user.

Clinical traits to consider include motor impairments and related symptoms that may influence motor planning and mental imagery, preinjury motor memory, and hemispheric dominance. Tailoring activities to align with familiar movement patterns can optimize results. Imaging findings can also guide task selection by identifying preserved brain regions and targeting specific lesions, helping to decide whether to promote contralesional or ipsilesional brain activity.

For inattentional problems like neglect, tasks involving continuous stimuli that shift toward the neglected hemispace or provide alerts can enhance awareness and engagement. For attention and concentration difficulties, discrete tasks with clear beginnings and endings are more beneficial. Continuous rhythmic activities may reduce focus and engagement, making structured tasks favorable for maintaining attention.

*We all have mental maps, [...] several meaningful tasks, and individuals select one of them. Each also implement this possibility of variability*.

[NE 4, workshop 1]

*The activities vary greatly, they’re very personal*.

[SS 10, workshop 5]

*There’s another thing that it should be done which is to analyze not only from a clinical viewpoint, but also with neuroimaging findings. [...] What area was spared or was it initially recovered after stroke? From that point it’s very specific to the person. Was the stroke more localized to the primary motor area only? Or somatosensory and motor? Or there’s pre-motor and other regions?*

[BE 1, workshop 6]

### Guidelines for Structuring BCI Interventions

#### Starting Levels

The key concepts for the initial levels focus on simplicity, facilitated by task segmentation, and align with motor development theories that prioritize gross motor skills before fine motor activities. This approach ensures that foundational skills are established before progressing to more complex movements.

Simple tasks benefit both mental imagery and the capabilities of EEG for capturing brain activity, as EEG’s limited spatial resolution makes straightforward tasks more effective for analysis. Considering motor imagination, movement simplicity helps maintain focus on the goal.

Participants noted that segmentation can facilitate MI, especially in the early stages, as visualizing complex tasks with multiple movement components is challenging. However, participants emphasized the importance of balancing segmentation with maintaining focus on the overall task, thereby preserving the connection to real-world movements. One suggestion was to complement segmented tasks with a video demonstrating the complete task to help reinforce this balance.

*Regardless of whether the patient is more or less able, the task should be as simple as possible in terms of imagery. [...] we want a sensorimotor pattern captured that is representative of that task. If the task varies too much, or if it’s fundamentally complex, I can’t create an activation pattern that represents that task.*

[NE 10, workshop 3]

*It’s important to start by segmenting, with very simple things, because every part of the movement is difficult to imagine. For example, holding a fork it’s very difficult to imagine because you must go over there, pick it up - each person picks it up in a different way - and keep holding it. So, starting off in stages and then gradually trying to add movements.*

[BE 3, workshop 6]

#### Dimensions for Progressing

Progression involves gradually increasing the difficulty of a movement task by adjusting task components, modifying parameters, or modulating technological features. Due to the challenge of visualizing movements with multiple components and their correct timing, adding components to a task should only occur after mastering previous movements, which may require time and be achievable over the long term, rather than in a single session. While increasing task difficulty through imagery is a form of progression, capturing distinct cerebral activation patterns with EEG can be challenging.

Imagining familiar movements was perceived as easier due to motor memory, so introducing less familiar tasks can effectively increase difficulty. Another method is modulating the threshold of imagination, where the intensity of brain signals required to elicit movement in the virtual reality environment is adjusted without changing the task itself.

Relating activities to real-world contexts can enhance perceived difficulty. Visual alterations and cognitive cues provided by technology can facilitate this sensation. Participants also suggested that increasing the movement’s range of motion could contribute to progression. Introducing dual cognitive tasks or increasing feedback stimuli during MI could raise task difficulty by adding cognitive load and making the task more challenging.

*That’s not to say that you can’t do something more sophisticated. Some people have done decoding, but it’s not easy [NE 3: But it’s not easy]. It’s true that if you think about various movements, different segments of the upper limb are located in slightly different regions, but the imagery patterns are typically a little blurred.*

[BE 1, workshop 6]

*I think that initially we should start with more familiar tasks and as activation improves, possibly as a matter of motivation, novelty and stimulating more cortical representation, we’ll move on to new tasks.*

[NE 5, workshop 2]

#### Progression Criteria

Progression criteria are an indicator that a user is ready to advance to a more challenging task level. Discussed criteria included human assessment and technology-assisted feedback. The decision to progress can be made collaboratively by the stroke survivor and the health care professional, according to the stroke survivor’s performance and insights. This approach aligns with the core principles of individualization and collaboration, fostering a shared decision-making process within the therapeutic alliance. Progression can also be time-based, occurring after a period of accurately performing the proposed task.

Gamification and scoring systems can guide progression, where accurate repetitions of movement imagery are tracked, and upon reaching a predefined number, the next level is unlocked. In addition, EEG can provide an objective measure for determining the ideal moment for progression. Participants suggested that, by monitoring the connectivity of different electrodes, its increase may indicate that the imagery activity is becoming more robust, representing a readiness to advance to the next level.

*Here you could also make a system of trying and counting successes of repetitions like in gaming, for example, with the user’s decision. [NE 3: But including the user’s decision]*.

[BE 2, workshop 6]

*One possibility is to look at the connectivity of the EEG [BE 1: Yes] because, in principle, if the connectivity is too great between several zones, there will be greater recruitment because there is a lot of effort being made*. *As connectivity becomes more concentrated [NE 3: Exactly], it means that the person is optimizing the task they are training for. Connectivity with the EEG can help. With this type of set, it will be electrode-based connectivity, you can’t do the other part, which is regional.* [BE 2, workshop 6]

#### Session Duration

One key aspect discussed regarding the design of BCI-based MI interventions was the optimal session duration. The ideal length should balance achieving meaningful neural changes with maintaining motivation for effectiveness and sustained engagement. Participants suggested that sessions should not exceed 20 minutes, citing the difficulty of maintaining attention levels due to the repetitive and cognitively demanding nature of the tasks.

Neurorehabilitation experts reported a higher recurrence of fatigue during ULR compared to other interventions, indicating that UL recovery is more demanding. Fatigue emerged as a reliable, person-specific indicator to determine the appropriate end point for each session or the need for breaks. Rest is beneficial for promoting recovery processes that help the brain assimilate new skills and consolidate gains, optimizing the learning process. The importance of integrating BCI therapy with conventional therapy was emphasized, with participants stating that, respecting patients’ fatigue and tolerance levels, performing BCI intervention before conventional therapy could yield better benefits, acting as a preparation for motor execution.

*Usually, it’s always the activities with UL that cause more fatigue [...] Or, they can disperse more attention*.

[NE 2, workshop 1]

*We have to assess each person and understand how long the patient can hold out. Some can take 15 minutes, others 10*.

[NE 12, workshop 6]

*Because in practice we’ll be teaching the brain to think about motor skills again. Therefore, for learning, we need breaks, we need repetition. So, it’s more advantageous to do repetitions and perhaps shorter cycles than doing a one-hour session.*

[BE 1, workshop 6]

### Key Factors Influencing Motivation

#### Variability

Variability was defined as the variation of a task without altering its inherent nature or difficulty. Stroke survivors expressed that continuous repetition negatively impacts their motivation, causing feelings of monotony and making it difficult to maintain attention and focus on the task. Variability was proposed as a means of “repeating without repetitiveness,” breaking monotony and aligning tasks more closely with real-world contexts where pure repetition is rare. However, participants cautioned that variability should be introduced progressively to avoid disrupting focus.

Participants suggested that alternating the type of feedback could help break the cycle of demotivation and inattention. The effectiveness of offering varied, tactile, visual, and auditory stimuli was explored as a means to provide novel information and maintain user engagement. Incorporating variability in visual content (eg, object color) was frequently mentioned as a key strategy. In addition, introducing fictional elements, characters, or well-known celebrities could enhance task variability and further engage participants.

*For some people can be demotivating doing the same task over and over again*.

[SS 17, workshop 5]

Here, although it’s the same manual clamp for the car, it has different abduction and external rotation components. But to put several doors: house or shop doors with the same movement, that’s repeating without repeating. It varies according to the context, but the task is the same.

[NE 2, workshop 1]

#### Gamification

Creating gamified VR scenarios by introducing game-like elements, namely, scoring, helps reduce the focus on the movement itself, facilitating execution. This shift in focus alleviates negative emotions typically associated with challenges in arm movement. Promoting VR gamification supports repetitiveness, which in turn promotes the learning process.

Participants noted that assigning scores to task performances could leverage this playful atmosphere. Since individuals may respond differently to gaining or losing points, participants suggested that the scoring system could be adjusted by the professional, representing an additional method for personalizing the intervention.

*[when playing] I would relax and practise the movements in a pleasant and emotionally unburdened way*.

[SS 13, workshop 5]

*If people lose, they can become demotivated. You can always do gamification to win badges, medals, points, anything and never lose*.

[SS 10, workshop 5]

#### Activity Demand

Participants proposed that the intervention program should include a graded system for task selection with an ongoing process of monitoring and adjustment of activity demand. Adjustments may involve either increasing or decreasing task difficulty and have the goal of maintaining an optimal level of difficulty, based on each stroke survivor’s capabilities. This approach aims to prevent discouragement from tasks perceived as too difficult and demotivation from tasks considered too easy.

Using a rationale based on small incremental steps would allow for the inclusion of intermediate levels, preventing tasks from being excessively easy or overly challenging.

*I think that [...] the activity can be graded, and the therapist himself can define what kind of activity can be done and the difficulty, considering the patient*.

[NE 3, workshop 1]

*It’s roughly this game theory—you want to be in the sweet spot between the difficulty of the task and your competence […] The difficulty must be such that I can overcome it. If I can’t, you adjust the difficulty. That’s how you progress. And that’s what best keeps the participant’s attention and engagement*.

[BE 1, workshop 6]

### Technology Features

#### Set-Up Considerations

Regarding the physical position of the patient, 2 key variables should be considered, namely comfort and the promotion of postural stability. Appropriate back support and, if possible, support for the UL on a table or equivalent surface should be provided. This setup promotes an external contextual organization that could facilitate greater focus on brain activation.

When providing instructions for the task, previous action observation through video was considered a helpful strategy, particularly for stroke survivors with language impairments. In addition, this approach enhances the precision of MI-related brain activity.

*To me it makes sense that the person is comfortable in a comfortable position and that there won’t be confounding variables around while he is doing this, right? [For example] The person being unbalanced and trying to manage what is the real body with the virtual body [would be confusing or distracting*].

[NE 10, workshop 3]

*[...] to ensure that the task is recognized, not only in its context, but also in its objective, having an example of a person reproducing the movement would be important because it has cortical representation [...] which would give a better feedforward*.

[NE 2, workshop 1]

#### Providing Feedback

The immersive VR environment was viewed as beneficial for creating enriched contexts that facilitate neuroplasticity through meaningful, multisensory, and enjoyable stimuli. However, for individuals with attention deficits, perceptual disturbances, or sensory impairments, overstimulation could have negative effects. This highlights the need for personalized feedback to adjust the quantity and intensity of stimuli to meet individual needs.

Regarding visual feedback, participants noted that human-like features are more interesting and meaningful to users, with recommendations to match skin tone and clothing. The use of “extraordinary” characteristics may also serve as a motivational factor. Visualizing movement through an avatar can aid in perceiving that movement, improving bodily awareness. In addition, related to body schema, the presence of both ULs within the field of vision could be beneficial. For those who may not tolerate virtual reality glasses, using a large screen was proposed as an alternative to induce a sense of immersion.

Considering haptic feedback, stroke survivors highlighted the importance of tactile stimulation, in addition to visual engagement, particularly vibration, which can vary depending on the task and scenario. The use of transcutaneous electrical nerve stimulation was highlighted for promoting cortical reorganization.

In addition to observing movement in VR and receiving haptic feedback, stroke survivors expressed that experiencing the movement, including proprioceptive feedback, would be more meaningful. It was suggested that a device (eg, a robotic glove) either simultaneously or after should facilitate actual hand movement to avoid muscle tone increasing by trying to move.

*I think the strategy here is adding stimuli or cues that the brain can pick up to support the final intention. This question of the shark is excellent, because in practice I’m going to have extra motivation. By activating the amygdala I’m going to look for other resources to carry out the task*.

[BE 1, workshop 6]

*The only thing to do is open and close the glove, when we think about opening and closing. And that would solve the haptic feedback [SS 9: Exactly! It’s the moment our brain recognizes...] Because our hand doesn’t close, but if I’m thinking about closing and the hand closes, it might be interesting*.

[SS 10, workshop 5]

## Discussion

### Main Findings

By undertaking a multiperspective qualitative study, this study aimed to explore the insights from neurorehabilitation experts, stroke survivors, and biomedical engineers. From the analysis of the results, 6 themes emerged with key recommendations for the design and implementation of a MI-based VR-BCI tool for UL poststroke rehabilitation, which can significantly enhance its effectiveness. By integrating the perspectives of diverse stakeholders, the study informs the development of MI-VR-BCI tools that are not only technically sound but also user-friendly and tailored to the needs of stroke survivors. The individualization of treatment throughout the intervention process, associated with multidisciplinary teamwork and shared decision-making, emerged from the study as important, representing also essential pillars of the patient-centered approach [69]. It is a recommendation in line with international guidelines for UL stroke rehabilitation and provision of health care [11,29,30,70].

This interdisciplinary collaboration has the role of providing comprehensive health care and establishing treatment goals and planning in a shared manner, having in mind the patient’s functionality [11,70]. Goal establishment must consider that individual needs vary, influenced by stroke type, symptoms, and the individual’s cultural and psychosocial circumstances, and must be well-documented, specific, and challenging [70]. While specialized counseling and orientation by a professional when using VR is seen as important [71], the autonomy support (providing individuals the ability to control their own behavior) also enhances UL performance and promotes the use of the more affected UL after stroke [72].

According to recommendations for stroke recovery trials, a holistic, biopsychological assessment must be performed [73,74], encompassing the 3 dimensions of the International Classification System (ICF) [75], namely body functions and structure (impairment), activities (limitations), and participation (restriction). According to international stroke assessment guidelines, the Fugl-Meyer Assessment, which evaluates impairment, should be complemented by the Action Research Arm Test to assess the activity dimension [74]. Integrating instrumental evaluations, such as inertial motion sensors, can further enhance the accuracy of UL impairment assessment [76] and provide complementary insights into patient-reported outcomes related to activity and participation [11]. The assessment of the quality of movement performed (another important aspect in the context of investigation of UL-oriented interventions) might be achieved through performance assays (such as 2D reaching, finger individuation, and grip and pinch strength) and a 3D functional drinking task [77]. These recommendations shall be taken into consideration since, despite them, most research primarily addresses structural factors, with only a portion, including activity [73], a pattern also found in BCI research.

Following the recommendations from our participants, it is essential to establish clear criteria for effectively assessing MI capability [28,29], and neuropsychological aspects like attention and concentration [22]. Considering cognitive functions, Kruger et al [78] emphasize the importance of working memory capacity for MI, as it depends on the ability to retrieve stored sensory information about a specific movement from long-term memory, transform it, and maintain it within working memory. There was a divergence of opinion among participants concerning the inclusion of patients with cognitive impairments, specifically those affecting attention, perception, and mental imagery capacity. In an observational study on influencing factors on MI after stroke, visual-spatial skills were correlated with MI visualization quality, indicating that altered visual-motor skills may impact both MI and movement [26]. Rienzo et al [34] found similar evidence but stated that, according to the evidence found, visuo-spatial and perception impairments seem to have more influence on MI tasks of an implicit and not explicit nature, since implicit MI involves manipulation of mental representations of affected body parts. In agreement, attention and spatial abilities were found to be performance predictors for MI-based BCI, together with the users’ relationship with the technology (such as computer anxiety and sense of agency) [79].

Considering neglect, potentially leading to perceptual impairments and visuomotor deficits [80], there is evidence showing that not only contralesional UL visuomotor MI tasks reduced some neglect symptoms (manifested through copying and drawing tasks) but also enhanced UL sensation [81]. These clinical traits are more commonly associated with right hemisphere lesions, including parietal lobe and fronto-parietal pathways [80], and parietal stroke survivors are often excluded from MI studies [82]. Even so, this lesion location alone, or the presence of neglect, does not necessarily predict MI capacity, as neuroplasticity may allow other brain regions to compensate for the damaged areas [34,83]. These findings should be carefully considered, as factors such as neglect may not justify excluding stroke survivors from studies or clinically implementing MI-VR-BCI that involves explicit MI, and patients can possibly benefit from the intervention. However, these factors may influence the intervention outcomes and design, including the MI training required and the selection of task difficulty.

UL functionality should also be considered in decision-making regarding training protocol, as it directly impacts motor execution that correlates with MI capacity [26]. Initial severe UL impairment (Fugl-Meyer Assessment-Upper Limb ≤30), significant hand spasticity (Modified Ashworth Scale-Hand ≥level I+), and presence of aphasia have been associated with less favorable distal UL recovery after MI-based BCI intervention [84]. Stroke location may also play a role regarding intervention potential, as supratentorial strokes and lesions in the pontine region are associated with poorer UL recovery (with the latter linked to severe disturbances in both sensory and motor functions) [85]. Basal ganglia lesions are found to particularly disrupt frontal lobe connectivity and motor planning [85], possibly negatively influencing rehabilitation outcomes. As corticospinal tract integrity is a strong biomarker of sensorimotor function and motor recovery [86], it may be considered for establishing patient profiles and intervention conditions, with higher corticospinal tract integrity and some degree of residual muscle control possibly particularly benefiting from combined EEG and electromyography systems, enhancing BCI performance [87]. Interestingly, although sensorimotor function may be a predictor for success in intervention, in cases of severe compromise of the corticospinal tract, MI may be particularly beneficial by optimizing effector-independent learning mechanisms, relying on additional projection systems (ie, the corticoreticular tract) [88], and enhancing connectivity between the sensorimotor network and other brain networks [89]. To tailor clinical decisions regarding rehabilitation options and enhance prediction of therapy response, it will be important to assess white matter disconnection patterns (disconnectome), besides clinical presentation and stroke location [88]. In addition, stroke stage must also be considered for decision-making on optimal training protocol. While effective in both subacute and chronic phases, BCI shows greater effect sizes in the subacute stage, and efficacy may change according to BCI feature combination (for a meta-analysis [90]). Chronic phases with smaller effects might require adjustment of intervention dosage, with Salvalaggio et al [91] suggesting that effective UL interventions in the chronic phase must be delivered in a minimum of 10 hours if augmentative therapies are used, while priming interventions require higher doses (between 10 and 30 hours).

When considering task selection and execution, rehabilitation principles to augment motor control and restore sensorimotor function recommend the training to be task- and context-specific, goal-oriented, repetitive, meaningful, and function-based [29,30]. Accordingly, participants in our study mentioned the importance of the activities being based on ADLs as they represent more meaningful and useful activities when transferred to real needs. These results are in line with those collected by Isbel et al [71] in a study of a similar nature, with participants stating that the use of daily encountered activities is not only more relatable but also more motivating. The recommendation of context-specific training is also a perspective that VR can leverage by allowing the representation of different scenarios and an adequate selection according to stroke survivors’ needs. Although the MI-VR-BCI paradigm does not involve motor practice, as it shares principles with scientifically supported task-practice interventions [29,30,92], its principles may be useful for task design [93].

This recommendation is significant because, although current VR development guidelines consider the importance of task relevance [94], a meta-analysis conducted [7] on the design characteristics of BCI paradigms revealed a gap regarding the tasks performed. Most common BCI tasks involved motor attempts or imagination of grasping and extending the fingers, multijoint movements, or individual finger movements, with only a few including ADLs. Aprigio et al [35] provide an overview of tasks used in conventional MI, noting that the most commonly performed activities are rooted in daily life functions. Interestingly, these align closely with the activities our participants identified as significant. Thus, it is crucial to steer BCI task development toward activities that mirror daily life functions.

When considering the practice of bilateral or unilateral activities, the effects of each activity modality remain unclear [8,30,32,95], and evidence shows that bilateral activities appear to have more impact on function-related outcome measures and unilateral activities have more impact on activity dimension [95-97]. In line with the participants’ recommendations, clinical guidelines [29] recommend that both bilateral and unilateral arm training must be considered, taking into account the task and level of impairment.

Regarding intervention structuring, and in line with the recommendation to grade activity difficulty to match the stroke survivor’s abilities, Proulx et al [55] emphasize the importance of tailoring tasks to individual progress. The authors advocate for adaptable difficulty levels and appropriate task demands, noting that intense active practice requires continuous attention and focus from the stroke survivor. Keeping task demands appropriately challenging requires continuous adaptation and progression [30], which in turn supports both motivation and motor learning [54] and may facilitate the experience of flow state during MI-based BCI tasks [98]. Accordingly, Di Rienzo et al [99] found that low-demand tasks can induce fatigue and boredom, reinforcing the need to optimize task difficulty. Poveda-García et al [26] suggest aligning task difficulty with motor function, as motor function correlates with MI capacity. To support this individualized balance, BCI systems may incorporate performance accommodation mechanisms (PAMs), which dynamically adjust task difficulty based on the user’s performance, helping to maintain engagement and motivation. Jochumsen et al [100] compared various PAMs, including augmented success, mitigated failure, and input override. The authors identified that users with low BCI control experienced greater perceived control when assisted by PAMs, while those with high BCI control reported decreased perceived control and reduced performance when assisted. Accordingly, selection and implementation of PAMs should consider user performance in real time but account for individual differences in BCI control.

While task complexification was identified by the participants as a key method for progression, using EEG to capture brain activity poses challenges due to its high temporal resolution but poor spatial resolution [101]. Although BCI systems can record activity from cortical and subcortical networks, the detail and extent of the captured information largely depend on the signal acquisition method. MI activity is most effectively detected in the premotor cortex, primary motor cortex, and supplementary motor area, which are also activated alongside the basal ganglia and thalamus [102]. However, because EEG is a noninvasive method, it can only reliably capture signals from cortical areas, whereas intracortical electrodes are required to access activity from the basal ganglia and thalamus [16]. The increasing complexity of tasks, combined with the varying accuracy of MI, may not be fully captured by the recorded signals. This raises concerns that imagining an activity inaccurately could strengthen neural circuits that hinder effective motor learning. These considerations highlight the critical importance of accurately measuring brain activity and ensuring that mental imagery of tasks is closely aligned with the intended motor objectives.

As briefly mentioned by our participants, using high-density mapping could increase spatial resolution but result in a difficult signal-to-noise ratio across channels [103]. The challenges associated with data acquisition, processing, and pattern recognition in MI-based BCI interventions are increasingly being studied in order to overcome the challenges associated with using EEG and improving accuracy [104]. Saha et al [16] theoretically explore the addition of other source-capturing signals like functional near-infrared spectroscopy or magnetoencephalography in a complementary way to EEG to boost classification performance. The recommendation to create more complex scenarios by increasing cognitive demands (eg, by introducing additional visual or auditory stimuli) aligns with previous recommendations [55] and offers an alternative means of raising task difficulty. This method emphasizes cognitive mechanisms rather than relying solely on motor challenges, also recruiting dual-task mechanisms, as stated by participants.

Using connectivity as a coadjuvant progression criterion to assess MI efficacy aligns with findings from the study by Bagarinao et al [105], which indicate that MI expertise leads to more targeted recruitment of motor networks and higher brain activation intensity. Regarding the integration of MI-VR-BCI into clinical practice, participants stated it should complement conventional therapy, which is in line with the findings of Kruse et al [22] with MI acting as a priming tool with positive influence on impairment and activity outcomes in patients with stroke [106].

Motivation levels directly influence the process of ULR, posing as a fundamental aspect of intervention planning [107]. While repetition is essential for neuroplasticity and learning, requiring substantial practice volume and intensity, it can present challenges for sustaining patient engagement and motivation. In line with our recommendations, participants from Isbel et al [71] also stated that motivation can be enhanced through realism, gamification, scoring, and feedback. The usage of variability, besides having a positive impact on motivation, is a principle associated with optimized motor learning [108]. Moreover, gamification is a strategy with proven results in rehabilitation, associated with higher levels of motivation [109]. Its addition to VR shows improvements in the overall effect in comparison with only VR [36]. Gamification has been increasingly explored as a strategy to optimize MI-based BCI training protocols and outcomes. By enhancing motivation and engagement and reducing the monotony often associated, gamification may improve BCI performance (average classification accuracy of 74.35%) and support adherence and sustained participation in the intervention [110]. A recent systematic review [111] explored gamification in MI-based BCI training, highlighting the positive impact of elements, such as feedback, avatars, assistance, and social interaction, on both performance and user experience. However, most studies lacked rigorous designs, often missing control groups or relying on small samples, making it difficult to draw robust conclusions about the impact of individual game elements on effectiveness.

Regarding feedback customization recommendations, Proulx et al [55] state that treatment design for individuals with stroke must consider feedback delivery parameters, task complexity, and sensory deficits. They argue for combining various modalities of augmented feedback tailored to each individual condition, rather than a one-size-fits-all approach. Accordingly, the addition of multiple types of feedback may enhance performance and motivation and minimize frustration in healthy participants [112]. However, in patients with stroke, symptoms such as sensory hypersensitivity, including hyperesthesia, or potential cybersickness must be carefully considered, and optimal conditions should be established, ensuring adequate levels of immersion and presence to optimize the benefits of VR. When considering multimodal stimulation, feedback adjustment, and instruction modality, it is important to recognize that the relevance of different feedback types may vary depending on the nature of the task, its significance to the intended action, and the distinct brain areas and cortical networks they activate [78].

In the absence of proprioceptive feedback, visual feedback becomes particularly salient, making it a critical factor for MI-VR-BCI designs. Consistent with the statements of participants in our study, who expressed a preference for avatars with humanoid characteristics, several studies have explored the benefits of anthropomorphic design [44,47]. Through an experimental paradigm involving healthy participants, Alimardani et al [47] identified that using humanlike features raises the sense of agency, ownership, and embodiment. These findings are complemented by Bashford and Mehring [113], who showed that observing movements congruent with the imagined task can enhance the sense of control over the rendered body—even in the absence of accurate, real-time BCI performance. Although MI-based BCI performance may not differ between humanoid and abstract avatars, humanoid design can significantly increase users’ sense of ownership and agency [44]. By reproducing a more faithful representation of the imagined movement and aligning with the users’ real-world sensorimotor expectations, higher excitation of motor processes might be induced, which, in turn, may promote more efficient connectivity and aid the learning process [47]. Considering MI as not only motoric but also motivated by multisensorial movement conception and predictions [114,115], BCI researchers and designers might leverage multisensorial nature and VR environments to elicit a MI response.

Feedback in BCI systems is a multidimensional construct that encompasses more than just modality (eg, visual, haptic, or proprioceptive cues). One critical dimension is feedback granularity, which refers to the level of detail and continuity of feedback over time, encompassing aspects of spatial and temporal congruency, which are closely interrelated [116]. In discrete feedback, the system responds only when MI activity surpasses a set threshold, providing a brief, event-based signal. In contrast, continuous feedback offers real-time information about user performance, with the participants of Kjeldsen et al [117] showing a preference over discrete feedback due to a better sense of agency and movement notion. Despite this, in the same study, it was only possible to establish a correlation between higher agency and performance in the discrete feedback condition. Another key dimension is feedback bias, typically described as positive, negative, or neutral. Positively biased feedback, which rewards users regardless of classification accuracy, can temporarily enhance embodiment and MI performance [118]. Conversely, negative feedback within embodied VR, presenting movement with the contralateral side of the intended imagery during unsuccessful attempts, may impair the sense of agency and hinder MI learning [47]. When comparing contingent feedback with independent feedback through BCI-functional electrical stimulation (FES) in patients with chronic stroke, MI-contingent feedback led to significantly greater improvements in wrist extensor strength, range of motion, and functional connectivity in the affected hemisphere [20]. In addition, Maier et al [108] state that rhythmic cues, explicit feedback (knowledge of results), and implicit feedback (knowledge of performance) are neurorehabilitation principles to consider when developing interventions to elicit motor learning in neurorehabilitation. Importantly, the effectiveness of feedback strategies appears to be performance-dependent, underscoring the need for personalized feedback planning that considers both clinical characteristics and individual BCI proficiency.

A key difference between motor execution and MI is that the latter lacks sensory feedback due to the absence of movement [34]. Motor execution–based BCI interventions have demonstrated higher efficacy in UL function rehabilitation [17,23]. Adding proprioceptive feedback through externally facilitated movement can enhance neural engagement and promote more effective motor learning and recovery. When considering the addition of proprioceptive feedback, meta-analyses comparing BCI intervention designs favor the use of FES for improving UL function [17,23]. Usage of neuromuscular electrostimulation is also recommended by stroke rehabilitation guidelines [11], particularly FES to reduce motor disability and improve function in both acute and chronic phases of ULR [30].

Considering technology implementation, several key steps are essential. To support smoother adoption and informed engagement, both health care professionals and patients must receive adequate education about the MI-VR-BCI system [119]. It will also be important to establish what the neural mechanisms underlying BCI effectiveness for stroke rehabilitation are [120]. In addition, standardizing protocols and assessment timings and using validated clinical scales are recommended to ensure consistency in tracking progress, enhance decision-making, and improve comparability across patients and studies [11]. Based on participants’ input, establishing normative data from healthy individuals may further support clinical interpretation by providing benchmarks for MI quality, engagement, and neurofeedback responsiveness. In addition, when considering BCIs in clinical practice, clear ethical guidelines must be established and followed to ensure proper data management, privacy, and security [121].

This study addresses important gaps in the MI-VR-BCI field by shifting the focus from technical performance in controlled research settings with healthy participants to user-centered, clinically meaningful design. Grounded in the lived experiences and expertise of stroke survivors, rehabilitation professionals, and engineers, our aim is to bridge the gap between empirical findings—often based predominantly on healthy participants —and their clinical applicability. Our approach inverts the traditional trajectory for BCI development, from system- to patient-centered, prioritizing design and outcomes that align with clinical effectiveness and established international rehabilitation guidelines. In doing so, this study raises awareness of stroke-specific symptoms often overlooked in BCI development—such as sensory deficits, altered body perception, attentional challenges, and fatigue—and how these symptoms critically influence both user engagement and system performance. While we contribute to identifying profiles of eligible patients with stroke, we also propose multidimensional guidelines for the development of MI-VR-BCI interventions and adaptation strategies for those with more complex clinical presentations. Our recommendations may help accelerate the iterative design of MI-VR-BCI interventions, paving the way for inclusive, individualized, and effective systems that address a wider spectrum of clinical needs and are truly applicable to stroke rehabilitation settings.

Ultimately, this work may lay the foundation for a more unified framework that integrates clinical profiles, BCI performance, and user experience to optimize training protocols and enhance both learning and meaningful rehabilitation outcomes.

### Recommendations

A list of recommendations was created for the development and implementation of MI-VR-BCI systems (Textbox 1), grounded on the results of the workshops and informed by international guidelines for poststroke rehabilitation.

Textbox 1. **Recommendations for the development and implementation of motor imagery (MI), virtual reality (VR), and brain-computer interface (BCI) technologies.**

**R1: Importance of patient-centered approach**

MI-VR-BCI interventions must be conducted through a patient-centered approach, based on individualized preferences, needs, and goals of the user, by an interdisciplinary team.

- Patients must be part of the decision-making processes throughout the intervention course.
- A balance between autonomous and supported work must be promoted.
- Interpersonal relationships with the professional care team positively influence upper limb rehabilitation by helping to deal with feelings of frustration and discomfort.

**R2: Clinical evaluation and patient selection**

Selection criteria must include upper limb impairment, cognitive, and communication assessment. Clinical traits, such as MI capacity, neglect, and depression must be assessed since they might influence intervention outcomes.

- All 3 International Classification System dimensions must be considered for a comprehensive evaluation.

- Upper limb impairment severity might influence patient adherence to MI-based approaches and should be considered in patient inclusion and task selection.
- Attentional and perception impairments might influence MI capacity and feedback processing, possibly requiring specific MI-VR-BCI adaptations.
- MI-VR-BCI interventions should prioritize inclusivity by incorporating appropriate adjustments to enhance applicability, thereby extending usability to a broader range of patients with stroke.

**R3: Recommendations for task design**

A variety of tasks must be available for selection to ensure meaningfulness for the user and suitability to clinical traits. In addition, tasks should preferably be based on ADLs, including unilateral and bilateral tasks.

- Unilateral and bilateral tasks engage different neurological activation patterns in the brain, and both should be used to maximize clinical outcomes.
- Task’s typical performance must be considered when designing the task (eg, unilateral or bilateral, symmetry and asymmetry, laterality).
- Movements involving the upper limb in a global and integrated manner may evoke more comprehensive brain activity that closely resembles patterns associated with daily life tasks.
- The preferred type of movement and its suitability may be influenced by the level of upper limb impairment.
- Neglect-related visuospatial impairments might benefit from continuous tasks toward the neglected hemispace.
- Body schema and visuomotor field impairments must be considered and might require specific adaptations to guarantee that both hands are seen and perceived.

**R4: Guidelines for structuring BCI intervention**

Interventions must be structured by different progressive levels, starting with simple, gross movements and adding complexity through additional movement features, cognitive demand, or MI difficulty.

- Movement simplicity and segmentation aid both MI and signal acquisition.
- Although segmentation might be beneficial, it is important not to lose sight of the overall task. This could be addressed effectively using video.
- Signal acquisition during movement complexity progression can be challenging; however, verifying the accuracy of mental practice is important, as it may influence the reinforcement of appropriate motor schemas.
- Session duration must consider patients’ tolerance and fatigue; the inclusion of breaks or rest periods might improve attention and engagement.

**R5: Key factors influencing motivation**

Task variability, gamification elements, and task demand adequacy should be used to sustain optimal levels of motivation.

- To introduce variability, the preferred option appears to be the visual characteristics of objects and the scenario.
- Variability should not disrupt the attention in the task.
- Gamification features should consider patient profile and optimal response, particularly leveraging scores, badges, feedback, avatars, assistance, and social interaction.
- Upper limb impairment severity might influence MI capacity and therefore, possible task demand. Performance accommodation mechanisms may help address this issue and should be adjusted to the user’s MI skill level and BCI performance.

**R6: Technology features**

Multisensorial potential of MI-VR-BCI must be effectively harnessed through the adequate adjustment of visual, haptic, and proprioceptive feedback modalities to the patient.

- Ensure the patient’s comfort and stability.
- Consider introducing feedback gradually, based on the patient’s attentional capacity.
- Allow the intensity of feedback and its parameters to be adjusted according to the patient’s tolerance.
- Proprioceptive feedback might improve intervention perceived relevance and bring MI-based BCI outcomes closer to those achieved through motor attempt.
- Visual and haptic feedback variability may help reduce monotony in training protocols.
- The use of a first-person perspective, human-like characteristics, and immersive VR may increase embodiment and facilitate the learning process.
- Consider leveraging the scenario to induce the MI process seamlessly and naturally, reducing cognitive effort.
- Granularity and feedback must be considered and tailored to the patient’s BCI performance and individual responsiveness.
- Consider timing and spatial congruence in relation to real-world conditions and using MI-contingent feedback.
- Consider including FES to improve outcomes in upper limb impairment.

### Limitations

Although the sample’s multiperspective nature is a strength, participants’ limited experience with the technology, due to its innovative nature and lack of clinical implementation, represents a limitation. Despite efforts to address this through rich and elucidative activities, our study design and methodologies did not allow for full experimentation of the MI-VR-BCI paradigm, due to the logistical constraints and design methods used. Findings may be context-specific and influenced by individual participant experiences and knowledge. This reality suggests the need to validate the recommendations, using, for example, a nominal group technique. In that case, we suggest a balanced inclusion of diverse clinical neurorehabilitation experts and stakeholder representatives to capture a comprehensive and balanced range of perspectives.

Many of the recommendations suggested were based on a theoretical foundation and construct validity. Thus, it will be important to develop efficacy studies that can adequately isolate influencing variables and mechanisms of action, also helping to establish the validity of the recommendations through empirical paradigms.

### Future Research

Despite promising advances, current MI-VR-BCI technologies face several limitations that hinder their clinical translation. These include low signal-to-noise ratios and variability in EEG signal quality [122], challenges in accurately classifying brain signals and supporting multiple classes [123], lengthy and demanding calibration protocols [110,124], limited real-time adaptability [125], and reliance on fixed protocols that do not allow for progression [79].

To overcome these limitations, future research should focus on developing adaptive, patient-centered MI-VR-BCI systems that are aligned with international clinical guidelines and real-world clinical needs. This includes optimizing training protocols in terms of duration, progression, and cognitive load management to sustain engagement and motivation. Equally important is raising awareness among, and involving, health care professionals and patients early and continuously in the design and implementation process [119], to ensure that technologies meet real-world clinical needs, encourage adoption, and promote meaningful rehabilitation outcomes [126].

Future studies should analyze the interaction between different types and intensities of feedback (eg, immersive and nonimmersive VR and their effects on embodiment) and their relationship with task characteristics (eg, unilateral and bilateral activities), user profiles, and BCI performance. Considering stroke survivors’ characteristics, it will be important to stratify data according to stage of evolution, lesion location and network disconnection, level of impairment of somatosensory function, and perceptual and neuropsychological alterations. It is also important to establish the efficacy of gamifying training protocols and understand its relationship with motivation, engagement, and BCI performance. Studies should also establish the intervention’s effectiveness across ICF dimensions, both short- and long-term, and assess the cost-benefit ratio for different intervention features.

Technological solutions should evolve to better mimic real-life conditions and address stroke survivors’ clinical needs, balancing personalization with broader applicability.

### Conclusions

The MI-VR-BCI paradigm was very well received by participants in our study. The recommendations presented in this study for the development and implementation of MI-VR-BCI technologies aim to enhance their impact on ULR. Grounded in comprehensive consultation with all stakeholders, these recommendations will improve the technology’s relevance and potential efficacy. These recommendations should be taken into account in future studies in order to allow the analysis of the relationship between different characteristics of the stroke survivors, the MI task, and the technological features.

## Supplementary material

10.2196/71789 Multimedia Appendix 1 Supplementary material, including participants’ characterization questionnaires, workshop feedback questionnaires, workshop detailed structure, thematic analysis synthesis and summary with the relevant activities, movements and functions identified.

10.2196/71789 Checklist 1 COREQ checklist.

## Abbreviations

ADL: activity of daily living
BCI: brain-computer interface
COREQ: Consolidated Criteria for Reporting Qualitative Research
EEG: electroencephalography
ERD: event-related desynchronization
ERS: event-related synchronization
FES: functional electrical stimulation
ICF: International Classification System
MeSH: Medical Subject Headings
MI: motor imagery
MoCA: Montreal Cognitive Assessment
PAM: Performance Accommodation Mechanism
UL: upper limb
ULR: upper limb rehabilitation
VR: virtual reality
