# Supplementary material for: Recommendations for Combining Brain-Computer Interface, Motor Imagery, and Virtual Reality in Upper Limb Stroke Rehabilitation: Qualitative Participatory Design Study
Source: JMIR Rehabil Assist Technol. 2025 Oct 15;12:e71789. doi: 10.2196/71789 (PMC12527325; doi:10.2196/71789)
Supplement: Multimedia Appendix 1 [file rehab-v12-e71789-s001.docx]

**Multimedia Appendix 1**

Supplementary Data Methods

Patient Characterization (English Translation) - Page 2

Patient Characterization (Portuguese Original Version) - Page 3

Workshop Feedback Questionnaire (English Translation) – Page 4

Workshop Feedback Questionnaire (Portuguese Original Version)- Page 5

Table S1 Workshop Detailed Structure - Pages 6-14

Supplementary Results

Table S2- Thematic Analysis Synthesis – Pages 15-23

Table S3 - Relevant Activities– Page 24

Table S4 - Relevant Movements and Functions

**Patient Characterization Questionnaire (English Translation)**

| **Stroke patients** | |
| --- | --- |
| The following questionnaire aims to briefly identify each participant, to ensure that they meet the study's inclusion criteria and some diversity of participants. | |
| Please indicate your age: years | |
| Please indicate how many years ago you had your stroke (if you had more than one, indicate the first): years | |
| How would you classify your functional dependence?  □ Complete dependence;□ High dependence;□ Moderate dependence;  □ Mild dependence; □ Completely independent | |
| How would you rate your ability to move your upper limb? | |
| □ Unable to move;□ Has movement but is not functional for day-to-day activities;  □ Has movement that is mostly useful and functional for tasks day-to-day activities;  □ Shows movement that is moderately useful for tasks; □ Shows movement that is mostly useful and functional | |
| Indicate whether you have had previous contact with virtual reality:  □ No;□ Yes - before the stroke;□ Yes - after the stroke | |

**Patient Characterization Questionnaire (Original Portuguese Version)**

| **Utentes com AVC** | |
| --- | --- |
| O seguinte questionário tem como objetivo identificar sumariamente cada participante, para garantir que cumprem os critérios de inclusão do estudo e alguma diversidade de participantes. | |
| Por favor, indique a sua idade: anos | |
| Por favor, indique há quantos anos teve o seu AVC (se teve mais que um, indique o 1º): anos | |
| Como classifica a sua dependência funcional?  □ Dependência completa; □ Dependência elevada; □ Dependência moderada;  □ Dependência ligeiras; □ Completamente independente | |
| Como classifica a sua capacidade de movimento do seu membro superior: | |
| □ Incapaz de mover; □ Apresenta movimento, mas não é funcional para as atividades do dia-a-  dia; □ Apresenta movimento que é moderadamente útil para as tarefas; □ Apresenta movimento maioritariamente útil e funcional | |
| Indique se teve contacto prévio com realidade virtual:  □ Não; □ Sim – antes do AVC; □ Sim – depois do AVC | |

**Workshop Assessment Questionnaire (English Translation)**

| **Closed-ended questions (select one option from a scale of 1 to 6)** |
| --- |
| **How would you rate the experience of taking part in this workshop?**  **Negative 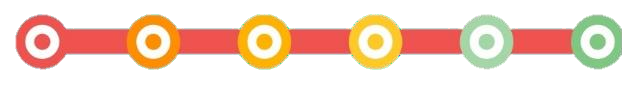 Positive** |
| **Did you consider this workshop to be productive for the purposes of this study?**  **Not at all productive 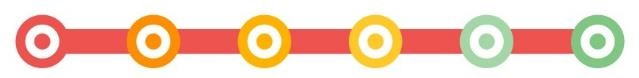 Quite productive** |
| **How would you rate the organization and moderation of this workshop?**  **Inappropriate 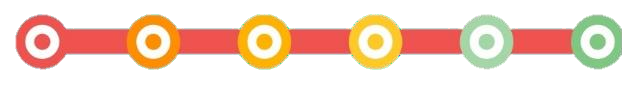 Adequate** |
| **Do you consider that participating in this workshop was a valuable use of your personal time?**  **Poor 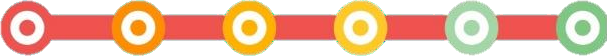 Valuable** |
| **Open questions:** |
| **What do you think was the most important topic discussed?** |
| **Do any relevant topics come to mind at the moment that weren't covered?** |
| **How would you describe your experience of attending this workshop?** |
| **In what ways could this workshop have been improved?** |
| **Other comments:** |

**Workshop Assessment Questionnaire (Original Portuguese Version)**

| **Perguntas de respostas fechadas (selecionar uma opção na escala de 1 a 6)** |
| --- |
| **Como avalia a experiência de participar neste workshop?**  **Negativa 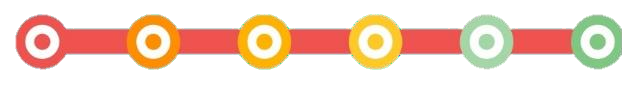 Positiva** |
| **Considerou este workshop como produtivo para os objetivos do presente estudo?**  **Nada produtivo 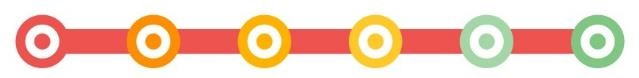 Bastante produtivo** |
| **Como avalia a organização e moderação deste workshop?**  **Inapropriada 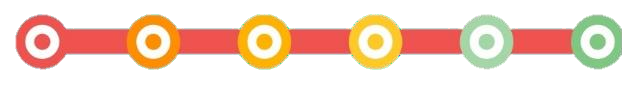 Adequada** |
| **Considera que a participação neste workshop foi uma utilização valiosa do seu tempo pessoal?**  **Pobre 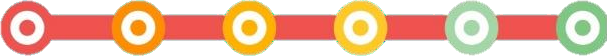 Valiosa** |
| **Questões abertas:** |
| **Qual foi, na sua opinião, o tópico mais importante que foi discutido?** |
| **Surgem-lhe, neste momento, tópicos relevantes que não foram abordados?** |
| **Como descreve a sua experiência na participação deste workshop?** |
| **Em que aspetos poderia ter sido melhorado este workshop?** |
| **Outros comentários:** |

**Table S1 - Workshop Detailed Structure**

| Wkp | Goals | Duration | Workshop Activities | Content |
| --- | --- | --- | --- | --- |
| 1 | Define the most important tasks and virtual environment characteristics for relevant brain activity | 90 min | Group discussion facilitated by the moderator, using image prompts in slides to illustrate the types of tasks mentioned. (75 minutes) | 1- Should the activity involve one hand (unilateral) or both hands (bilateral)? 2- If using both hands, should the movements be symmetric (same action) or asymmetric (different actions)? 3- Is it better to choose a familiar task or introduce a new/novel task? 4- Should the activity take place in a simple environment or a rich, stimulating environment? 5- Is a rhythmic, continuous task more appropriate, or one with a clear beginning and end? 6- Should the gesture be repeated in the same context, or performed in varied contexts? 7- Would it be more effective in a mundane, everyday situation, or a unique, extraordinary context? 8- Should the participant focus on just the hand, or the entire upper limb? |
| 2 | Define the most important tasks and virtual environment characteristics for relevant brain activity | 90 min | Group discussion facilitated by the moderator, using image prompts in slides to illustrate the types of tasks mentioned. (75 minutes) | 1- Should the activity involve one hand (unilateral) or both hands (bilateral)? 2- If using both hands, should the movements be symmetric (same action) or asymmetric (different actions)? 3- Is it better to choose a familiar task or introduce a new/novel task? 4- Should the activity take place in a simple environment or a rich, stimulating environment? 5- Is a rhythmic, continuous task more appropriate, or one with a clear beginning and end? 6- Should the gesture be repeated in the same context, or performed in varied contexts? 7- Would it be more effective in a mundane, everyday situation, or a unique, extraordinary context? 8- Should the participant focus on just the hand, or the entire upper limb? |
| 3 | Elect relevant adaptations and patient exclusion factors; Prioritize task characteristics defined in previous workshops | 90 min | Group discussion facilitated by the moderator using answer options to spark discussion. (75 minutes) | 1. Clinical Traits to Consider:  Absence of movement Pain Improper postural activity Incorrect spatial orientation Cognitive deficits  2. Task Relevance What types of tasks are most important or meaningful for the patient to perform in their daily life or recovery process?  3. Patient Assessment Tools Use standardized assessments to guide task selection and track progress: Barthel Index Functional Independence Measure (FIM/MIF) Action Research Arm Test (ARAT) Fugl-Meyer Assessment (Upper Limb Section) Motor Evaluation Scale for Arm in Stroke (MESUPES) Box and Block Test Motor Activity Log (MAL) – Assesses amount of use and quality of movement Active Range of Motion (AROM) Finger Motor Sequences  Other relevant assessments, as applicable |
| 4 | Delineate users’ priorities for intervention design and factors of motivation and engagement. | 90 min | Group discussion facilitated by the moderator, using : - image prompts in slides to illustrate the types of tasks mentioned - answer options to spark discussion; (75 minutes) | 1 – Experience with upper limb therapy. What factors help with: staying engaged; avoiding it; motivation; fatigue; frustration? 2 – Rehabilitation can become repetitive… What is your opinion? Possible solutions? Your own tricks? Therapist strategies? 3 – VR – previous experience: Has anyone tried it before? What do you think when you consider it? Putting on the headset? Using it in therapy? 4 – How to maintain motivation? Competition; challenge; fun; novelty; positive reinforcement; engaging experiences. 5 – BCI contextualization (Video showing) 6 – Selecting a specific movement: Should you use only the more affected arm? Should it be a simple, familiar movement?  7 – Which task would you prefer to train? (Hand grabbing a jug; hand grabbing an apple) (Child playing with soap bubbles; petting a dog) (Picking up a bottle; picking up a racket and playing tennis) (Opening a door; piloting a spaceship) (Cleaning windows; catching a ball)  Break-time scenes: Countryside landscape; black screen; forest; boat on a lagoon; city. |
| 5 | Assessing needs and goals for UL rehabilitation; Establishing preferences regarding the type of task. | 120 min | Small Group Discussion (3 participants) through World Caffe Methodology (12 minute discussion for each theme) followed by cardboard presentation to the whole group and discussion of the content to check for aditions and agreement. (50 minutes)  [15 minute break]  Group Discussion with image prompts in slides illustrating the types of task mentioned (45 minutes) | World Caffe Guiding Questions 1. What do you think is important in upper limb recovery? 2. “Today was really a good session”  a. what makes a session feel successful? b. And what are examples of less positive session experiences? 3. Motivation for Upper Limb Rehabilitation a. What factors contribute to motivation? b. What factors lead to demotivation?  Group Discussion Guiding Questions  Technology Experience 4. Has anyone ever had an experience with BCI or virtual reality and could you describe it? a. Do you think it could be a useful tool in rehabilitation? b. What aspects are most exciting or motivating to you? c. Do you see any downsides or challenges in using it?  Activity vs. Movement 5. The technology is based on performing an activity... a. Do you find it more interesting to train a single movement or to carry out a complete activity?  Feedback 6. What kind of information do you think is important to receive while performing a task? a. Feedback on performance vs. results b. Positive vs. negative feedback c. What kind of feedback do you prefer? (Visual, auditory, tactile)   Gamification  7. One of the possibilities of using technology is incorporating a game format, which allows, for example, scoring points. a. Do you see any positive aspects to using a game format? b. Do you identify any negatives or drawbacks? |
| 6 | Address points of divergent opinion; Discuss the technical feasibility of the recommendations made. | 145 min | Small Group Prototyping with Cardboard Guided by process-oriented questions to support each step. (60 min)  Simultaneous Technology Experimentation Each participant engages individually with the technology for 5 minutes in turn.  [15 minute break]  Group Discussion  Facilitated by the workshop moderator using guiding questions to prompt reflection and dialogue. (60 minutes) | Prototyping Guiding Questions  What information about the activity would be useful to display on the screen? Which aspects of the technology should the professional be able to adjust to allow for personalization? To make the activity more stimulating, what elements could be varied without changing the core activity?  Moderator Guiding Questions for Group Discussion  1. Clinical Traits and Inclusion/Exclusion Criteria for BCI-MI-VR Use Participants have discussed the influence of different clinical traits on using BCI-MI-VR technology. a. Considering the common clinical traits of stroke patients, can you identify inclusion and exclusion criteria for using BCI-MI-VR? Which criteria? (If possible, include assessment instruments) b. Are there clinical traits or diagnoses that require special attention or task adjustments but should not exclude the patient? (“consideration criteria”) c. How can we adjust task difficulty? That is, how do we make tasks harder (progression) or easier (regression)? d. Can EEG data be used to assess motor imagery capability?  2. Variability and Progression in Task Planning a. Participants have noted the importance of variability and progression when planning tasks. How can we distinguish between these two b. How can we create variability and progression when designing tasks for BCI-MI-VR in upper limb rehabilitation? At what points should we focus more on one or the other? (“progression criteria”) c. Does EEG reading allow detection of changes in movement speed or amplitude based on motor imagery signals? d. Can EEG data be used to shape treatment or inform decision-making about its course? (e.g., signal intensity) e. How complex can a task be, in terms of different components or sequencing? (How much complexity can EEG detect?)  3. Timing of Intervention. a. When do you think BCI-MI-VR should be introduced in a functional recovery program for the upper limb that also includes other therapies? b. What information should be provided to the patient when starting a BCI-MI-VR activity? c. Should the intervention occur before or after other therapies? d. Should it happen on the same day or on alternating days?  4. Feedback. Participants highlighted the importance of sensory feedback (e.g., visual, tactile, auditory), and also the importance of being able to adjust feedback intensity and complexity. a. Regarding visual feedback, what view is most important for the patient: first-person, third-person, or segmental? Is it possible to switch views based on needs and personalize? b. Patients mentioned the importance of feeling their arm move. Is it possible to provide proprioceptive feedback to simulate the experience of movement?  5. Task Laterality (Unilateral/Bilateral Activities). This topic generated mixed responses.  a. Considering unilateral and bilateral activities, do you see one type as more important? Which one would you start with?  6. Isolated Movement Training a. Do you see benefits in training specific, isolated physiological movements? Or alternatively, in segmenting and training more complex, combined movements? |

**Table S2 - Thematic Analysis Synthesis**

| Theme | Subtheme | Participant | Citation |
| --- | --- | --- | --- |
| Importance of Patient-  Centered Approach | Individualization of Care | (PwS 2, Wk 4) | “Eu acho que em primeiro lugar, temos que dar consciência de que cada AVC é único e diferente dos outros, assim como que cada processo de reabilitação é único e diferente dos outros, assim como nós pessoas somos únicos, diferentes dos outros, não é?” |
|  |  | (NE 13, Wk 6) | “Nós temos de colocar aqui o utente aqui no [Eng 1, Wk 6- Centro] no centro. Não é aqui à minha beira... mas é no centro” |
|  |  | Group Written Conclusions, wk 5 | P: Para vocês, o que é mais importante na recuperação do vosso braço? “*Participar com agilidade nas brincadeiras dos netos *Abraçar (dar aperto de mão) *Reconquistar a autonomia funcional;  *Mão funcional --> As tarefas diárias (comer, vestir, cuidados de higiene)" |
|  |  | Dialogue (Wk 5) | Pat 13- “Mas há esse negligenciar, como disse, e bem que é, é irritante porque nós não [Pat 15, Wk 5 -Não fazemos ideia que ‘tamos a fazer] não percebemos porque é que isso acontece. Pat 17- Sim e às vezes perdemos coisas que estão no tabuleiro [Pat 13, Wk 5 -Exato…] uma pessoa está só à procura do copo d'água. “ |
|  |  | PwS 15, Wk 5 | Por exemplo, eu fui afetada do lado esquerdo e eu a noção que tenho é que deste lado do meu campo de visão diminuiu. Eu, por exemplo, se for a conduzir e por isso é que a única mais tentei, nem tento e adoro é porque se eu for a conduzir, há a possibilidade de eu não conseguir ver, por exemplo, uma pessoa está a aproximar da passadeira. |
|  | Working as a Team | NE 13, Wk 6 | É importante também ter equipas [RE 3, Wk 6- Multidisciplinares] realisticamente falando multidisciplinares para todos percebermos um pouco o utente. |
|  |  | Group Written Conclusions, wk 5 | P: Quais as características de "uma boa sessão"? “*Capacidade de envolvência entre os terapeutas e os utentes (Capacidade de os terapeutas entrarem em nós) * Quando o terapeuta reconhece alguma progressão” |
|  |  | PwS 9, Wk 5 | Eu aprendo um exercício com a terapeuta (...) A seguir quero treinar, quero exercitar |
|  |  | NE 3, Wk 6 | “Tem que ser um trabalho em conjunto. Não pode ser a equipa de investigação isolada sem trabalhar com a equipa clínica.” |
| Clinical Evaluation and Patient Selection | Selection Criteria | Dialogue (Wk, 6) | NE 3 – “Até para a pessoa, se para ela, ela quer fazer ou não?” BE 1 –“Exatamente exatamente exatamente isso é a primeira coisa sempre. NE 3 – "Aí primeiro é a pessoa se ela quer fazer ou não, isso é o mais importante." |
|  |  | NE 13, Wk6 | “Se uma pessoa tiver desorientada nas 3 dimensões, dificilmente vai ter capacidade para fazer seja o que for. [RE 3- Exato, exato. RE 11 – Huhum.] E estamos a perder o nosso tempo no trabalho de reabilitação porque é muito complicado. Ou seja, nós temos que estabilizar, perceber qual é que é a parte cognitiva inicial da pessoa e depois trabalhar esta parte motora de forma intensa |
|  |  | NE 1, Wk 1 | (…) Há tão pouco (em estudos) esta capacidade de incluir alguns subgrupos de utentes que estamos a falar que, se calhar o desenvolver algo para essa franja de utentes (…) (iria ser) bastante relevante |
|  | Assessment of Influencing Factors | BE 1, Wk 6 | “O outro aspecto operacional que nós discutimos aqui bastante é, é a questão dos afetos (...). A questão da motivação. Se a pessoa estiver deprimida, provavelmente podemos estar a fazer todo o trabalho do, do mundo com quanto. Mas se não tivermos endereçar a questão afetiva…Se calhar o resultado poderá não ser tão positivo. Portanto temos de de atuar em em diferentes frentes. Portanto, poderá ser útil ou deveria ser obrigatório, provavelmente é, fazer uma avaliação psicológica prévia.” |
|  |  | Dialogue (Wk 6) | BE 1- "Não, não quer dizer que seja de exclusão, mas, mas provavelmente [RE 3- Dá-nos um score…] será necessário… [RE 3 - Exato..] Mais intervenção no treino de imagética que poderemos." BE 2 - "Sim, Exato." BE 3 - "Sim mas não como exclusão imediata…" BE 2 - "Exato, fazer um treino de imagética." |
|  |  | NE 2, Wk 3 | (...) quer à organização espacial, quer à capacidade de varrimento da imagem, da percepção do espaço e da noção corporal… Há aqui uma série de coisas que me parecem mais do foro cognitivo do que necessariamente motor, porque se de facto é só a imagética, (...) |
| Recommendations for Task Design and Selection | Task Characteristics | PwS 6, Wk 4 | Eu prefiro sempre treinos de coisas que façam sentido para mim na minha vida do dia a dia, (…) coisas sempre que sejam realmente práticas, que tenham alguma utilidade. |
|  |  | PwS 16, Wk 5 | É como, por exemplo, a realidade virtual para ir ao supermercado. Nós estamos, não estamos focados em tirar da prateleira, mas sim em…estamos a ver o que nós queremos meter no cesto, não é? Digo, eu. É um bocado por aí, não, não focar tanto no movimento, mas sim na ação. |
|  |  | NE 2, Wk 1 | É para para fazer com que reduzamos a concorrência em termos dos hemisférios cerebrais e eu diria que poderia ser unilateral. No entanto, sabemos que tarefas bilaterais favorecem, por vezes esta …ahh ahh… o movimento, o movimento ativo e a reabilitação. (...) Eu penso que convidaria a uma atividade unilateral e muito possivelmente procuraria inibir o ladro, o lado contralesional. |
|  |  | BE 1, Wk 6 | Mas aqui as 2 dá uma cue ao cérebro para, para imitar. De grosso modo, se a data science diz alguma coisa, ou da machine learning, é um transfer learning. Vamos transferir a aprendizagem de um membro para o outro |
|  |  | PwS 9, Wk 6 | Eu acho que bilateral faz todo o sentido. Exatamente para enganar. |
|  |  | NE 1, Wk 1 | Apelando à nossa realidade das atividades, (...) faz mais sentido, porque é efetivamente uma atividade assimétrica que nós fazemos desta forma. Mas, por exemplo, se eu tivesse uma imagem de carregar uma caixa com as duas mãos, como é uma atividade mais simétrica, também me apela mais ao meu registo e à memória motora que eu tenho da tarefa. Se calhar para mim não me faz muito sentido, estar a levantar dois copos. |
|  |  | NE 5, Wk 2 | São objetivos motores, e quando pensamos na mão temos três grandes objetivos motores, que é o alcançar, o agarrar e manipular. E que envolve o superior todo. E eu acho que as atividades deveriam envolver um membro inteiro, e não específica, porque se vamos focar só na mão, em termos de activação, vamos ativar predominantemente o circuito, claro, do agarrar, o da mão, não é? O que está relacionado pelo, pela mão, e estimulando menos o circuito do do do alcançar. |
|  | Personalized Task Selection | NE 4, Wk 1 | Todos temos mapas mentais, (…) em ter várias tarefas que tenham significância, e o indivíduo selecionar uma delas. Em cada uma delas, implementar também esta possibilidade de variabilidade. Isto no plano de sessão |
|  |  | PwS 10, Wk 5 | As atividades variam muito, são muito pessoais. Não é… |
|  |  | BE 1, Wk 6 | Há aqui outra coisa que eu que imagino que deva ser feita que é a analisar, não só do ponto de vista clínico, mas ter um suporte também imagiológico. (…) Que área é que eu que salvou? Ou que se conseguiu recuperar inicialmente? [RE 3, Wk 6-Neuroplasticidade…Exato.] E a partir daí tem de ser muito específico para, para a pessoa. Olha, sim… foi mais localizado área motora primária apenas, ou somato-sensorial e motora ou tem pré motora também e outras regiões. |
|  |  | NE 2, Wk 1 | Se vocês pensam em alguma atividade em que é tenha que surgir um varrimento na imagem ou uma chamada de atenção ao lado esquerdo para depois, então desenvolver a tarefa pensando e imaginando o movimento com com com o braço esquerdo. |
| Guidelines for Structuring BCI Interventions | Starting Levels | NE 10, Wk 3 | Independentemente seja um utente com mais capacidades, menos capacidades, mesmo para normais, a tarefa deve ser o mais simples possível, mais simples de em termos de imagética. (...) queremos um padrão sensoriomotor captado que seja mais ou menos representativo daquela tarefa. Se eu, se eu variar muito a tarefa ou se a variável, ou se a tarefa for complexa de base, eu não consigo criar, um, um padrão de ativação que representa aquela tarefa. Isto depois vai ser muito complicado... |
|  |  | NE 8, Wk 2 | (...) fazer esta decomposição, mas mantendo um foco em objetivo e à atividade, mesmo que estejamos a trabalhar apenas numa pequena porção, de maneira a manter todo o potencial de ativação da imaginação motora e a correlação depois com o movimento, mesmo que seja só o início do movimento (...) |
|  |  | BE 3, Wk 6 | Mas lá está convém começar segmentado. Mesmo com coisas muito simples porque cada, cada parte do movimento é difícil de imaginar. Por exemplo, agarrar um garfo. É muito difícil imaginar fazeres isto. Porque já tens provavelmente, ires até lá, agarrares, cada pessoa agarra de uma forma diferente e ficar a agarrar. Portanto, começar de forma faseada com movimentos à vez depois ir tentando gradualmente acrescentá-los [RE 11- Também acho que sim, é um bocadinho melhor] |
|  | Dimensions for Progressing | BE 3, Wk 6 | Claro que isto não pode ser feito numa única sessão, portanto, nós tínhamos pensado em termos de uma progressão, acho que nós tínhamos pensado mais a longo prazo e não na sessão em si. Portanto é longo prazo e lá está, tentar ir, acrescentando mais às, às sessões. (...) Portanto, fazer um primeiro movimento que neste caso seria, por exemplo, agarrar no garfo ou na faca e depois pronto ter um, quando a pessoa já tivesse bem, ter um segundo movimento. |
|  |  | BE 1, Wk 6 | Não quer dizer que não se consiga fazer qualquer coisa mais sofisticada. Há quem tenha já feito decoding mas mas não é fácil [RE 3- Mas não é fácil]. É verdade que aqui se pensar em vários movimentos tiver diferentes segmentos do membro superior estão localizados em regiões ligeiramente diferentes, mas os padrões de imagética tipicamente são um bocadinho blurred. |
|  |  | NE 5, Wk 2 | A novidade é motivadora, não é? Se for uma tarefa associada ao jogo mais motivador é (...) o pior é imaginar uma tarefa nova, ou seja, por um lado vai ser mais dificil imaginar(...) Eu acho que inicialmente também começar por tarefas mais familiares e à medida que a ativação vai melhorando eventualmente por uma questão de motivação, de novidade e de estimular mais a representação cortical, vamos passar para tarefas novas (…) |
|  |  | NE 7, Wk 2 | (…) aumentar os efeitos diastratores ou enriquecedor do ambiente, pode ser interessante, sem dúvida |
|  |  | BE 1, Wk 6 | Sim. Por exemplo, em termos de imagética, pode ser o, o threshhold que tu precisas ultrapassar para considerares que a imagética teve sucesso (...) Aqui será imaginar o movimento… aí... podes ter de de imaginar com maior intensidade. |
|  | Progression Criteria | BE 2, Wk 6 | “Aqui também se poderia fazer um sistema, por exemplo também funciona no gamming por exemplo, pode-se fazer um sistema de tentativa e de contagem de sucessos de repetições [AS- Humhum], mas depois com a decisão do utente. [RE 3, Wk 6- Mas incluindo a decisão do utente]” |
|  |  | NE 6, Wk 2 | Se fizerem isto tipo, jogo através do do scores, não é? Quando atingem determinado score de ativação, não é um determinado ponto, pode passar ao próximo nível que tem mais variabilidade. |
|  |  | BE 2, Wk 6 | Uma possibilidade é ver a conectividade do EEG [Eng 1, Wk 6- Sim] porque em princípio se a conetividade for muito grande entre várias zonas, haverá um recrutamento maior porque está a haver um grande esforço. À medida que a conetividade for mais concentrada [RE 3, Wk 6- Exato], significa que a pessoa está a otimizar a tarefa que está a treinar. A conetividade com o EEG poderá ajudar. Com este tipo de set, será uma conetividade baseada nos elétrodos, não dá para fazer a outra parte, que é a regional, não é? |
|  |  | BE 3, Wk 6 | Pela minha experiência, é que o terapeuta e o utente [Eng 1, Wk 6 – Os dois, exato], os dois em conjunto, é que definem “Olha, agora vamos passar para o nível seguinte” [Eng 1, Wk 6- É que definem] |
|  | Session Duration | NE 12, Wk 6 | Então temos que ter esse ajuste, só que se ajuste é como diz a [Eng 1, Wk 6- Ou fazer um treino] RE 13, tem que ser muito pessoa a pessoa e perceber… quanto tempo é que consegue aguentar. Algumas aguentam 15 minutos, outras 10 e depois já vem um pouco da parte do, do profissional [HS- Sim] que ele está a tentar balizar a intervenção |
|  |  | NE 2, Wk 1 | “Normalmente são sempre as atividades com o membro superior, as atividades que provocam mais fadiga. Não te sei dizer quanto tempo é que é o mais indicado, ou na clínica como é que normalmente as pessoas começam a bocejar mais cedo do que, quando é o trabalho de controlo postural, por exemplo.  Ou, ou podem dispersar mais…. A atenção.” |
|  |  | BE 1, Wk 6 | Porque na prática, aquilo que nós vamos estar a fazer é ensinar o cérebro a pensar novamente em motricidade. Portanto e para aprendizagem, precisamos de pausas, precisamos de repetição. Portanto, é mais vantajoso fazer repetições e ciclos, se calhar mais curtos do que vamos fazer uma sessão de 1 hora, é? |
|  |  | BE 2, Wk 6 | É que sem estas pausas não há depois os restantes processos bioquímicos ou eletroquímicos que estabilizam a neuroplasticidade que se conseguiu, o ensino, que se conseguiu a nível cerebral. Portanto, também é preciso haver estas pausas. |
|  |  | BE 3, Wk 6 | Sim, eu estou só a dizer que 20 minutos de seguida é impossível. 20 minutos de seguida a pessoa não consegue [Sim, depende da tarefa que se tenha] movimentos repetitivos. |
| Key factors influencing motivation | Variability | PwS 17, Wk 5 | E para algumas pessoas isso pode ser desmotivador, estar sempre a fazer a mesma tarefa. |
|  |  | NE 2, Wk 1 | Aqui, apesar de ser a mesma pinça manual para o automóvel, tem uma componente de abdução e rotação externa que é diferente. Mas colocar várias portas, ou uma porta de casa, ou a porta de uma loja, ou em que é sempre o mesmo movimento, isso é repetir sem repetir. Porque varia ao contexto, mas a tarefa é realmente a mesma |
|  | Gamification | PwS 13, Wk 5 | Eu descontraía e treinava os movimentos de uma forma agradável e sem carga emocional. |
|  |  | PwS 10, Wk 5 | Claro que existe, quer dizer ó… uma pessoa perde, pode se desmotivar. Pode-se fazer a gamificação sempre para ganhar uns badges, para ganhar os... medalhas e para ganhar os pontos e para ganhar o que for e nunca perder. Que é uma das opções da gamificação. |
|  | Activity Demand | NE 3, Wk 1 | Eu acho que (...) pode ser feito graduação da atividade e o próprio terapeuta é que define que tipo de atividade é que pode fazer, não é? E qual é o grau de dificuldade para aquele doente? |
|  |  | BE 1, Wk 6 | Portanto, é grosso modo, esta teoria de jogos. Tu queres estar no que se chama o sweet spot entre a dificuldade da tarefa e a tua competência. Portanto e tens de conseguir encontrar aqui um match. Portanto, a dificuldade tem de ser tal que eu consiga ultrapassar. Se eu não consigo, vais ajustando a dificuldade. Consigo? Vais assim progredindo. E é isso que melhor mantém a atenção e o engajamento do participante, do doente. |
| Technology Features | Set-Up Considerations | NE 10, Wk 3 | Para mim faz-me sentido que a pessoa esteja confortável, não é? Numa postura confortável e que não tenha outras variáveis parasitas à volta enquanto está a fazer isto, não é? A pessoa estar desequilibrada e a tentar gerir o que é o seu corpo real, com aquele corpo virtual. |
|  |  | NE 1, Wk 1 | acho muito importante neste tipo de terapias ah… esta organização do contexto e desta (...) esta posição inicial do indivíduo. Esta preparação para o movimento, independentemente de não existir um movimento, ainda, será muito importante. Lá está na preparação para, depois, a própria atividade. |
|  |  | NE 2, Wk 1 | (...) para garantir que a tarefa de alguma forma reconhecida, não só no seu contexto, como no seu objetivo, ter um exemplo de uma pessoa a reproduzir o movimento seria importante até porque tem representação cortical. (…) que daria um melhor feedforward. |
|  | Providing Feedback | BE 1, Wk 6 | Aqui acho que a estratégia, é estares a, estares a adicionar estímulos ou cues, pistas, nos quais o cérebro pode ir buscar, também. Acaba por suportar a intenção final. Portanto, é aqui esta questão do tubarão é excelente, porque na prática vou ter aqui uma motivação extra. A atívar a amígdala, fica aos gritos e vou buscar outros recursos para executar a tarefa. |
|  |  | PwS 10, Wk 5 | Portanto, será, a haver uma coisa destas, será ter parâmetros que o terapeuta poderá ajustar a cada utente. |
|  |  | NE 12, Wk 6 | Ah... e portanto, para além disso outra coisa que o Pat 17 também nos disse muito, que o afeta muito, é não ter a sensibilidade, não ter a parte dos estímulos. Então pensou-se também em associar à atividade essa... Como por exemplo os estímulos aaah... vibratórios, ter um.. Por exemplo, na alimentação ter diferentes texturas, se for carne, se for peixe, ter uma sensação diferente. Ah… se o copo estiver mais cheio ou menos cheio também ter esse feedback diferente por parte da aplicação. Se entornarmos também ter esse estímulo vibratório. |
|  |  | PwS 10, Wk 5 | Portanto, a única coisa que tem que se fazer é essa luva tem que abrir e fechar, quando nós pensamos em abrir e fechar [Pat 9, Wk 5- Exatamente.] E isso já ia resolver [Pat 9, Wk 5 - Eu penso que é isso mesmo] o ter um feedback ter um chama um haptic feedback, feedback háptico, do que a pessoa faz… [Pat 9, Wk 5 - Exatamente! É no momento em que o nosso cérebro reconhece, nós conseguirmos a..]. Porque a nossa mão não fecha, não é? [Pat 9, Wk5 -Exato] Mas se eu pensar em fechar e a mão fechar, é capaz de ser interessante. |

**Table S3 - Relevant Activities**

| Table S3 - Relevant Activities | |
| --- | --- |
| Dressing and undressing  - Tighten the shoelaces  - Buttoning a shirt  Eating & Drinking tasks  - Grab the food,  -Cutting  - Bring into mouth  - Grab a cup and drink   - Peal fruit  Hygiene         -To wash oneself         - Cut nails         - Put on cream         - Combing the hair | Hugging  Holding a pet (pet it)  Opening cans  Opening pills  Shaking hands  Sports activities  Swimming  Tidying the room  Driving  Clapping  Cooking (baking cakes)  Opening a window  Opening a jar |

**Table S4 - Relevant Movements and Functions**

| Table S4 – Relevant Movements and Functions |
| --- |
| - Reaching an object  - Grasping an object  - Manipulating an object  - Movements: Shoulder rotation and flexion  - Movements: Arm supination; open and closing the hand |
