# Supplementary figures and images for: Recommendations for Combining Brain-Computer Interface, Motor Imagery, and Virtual Reality in Upper Limb Stroke Rehabilitation: Qualitative Participatory Design Study
Source: JMIR Rehabil Assist Technol. 2025 Oct 15;12:e71789. doi: 10.2196/71789 (PMC12527325; doi:10.2196/71789)

**COREQ Checklist**

**
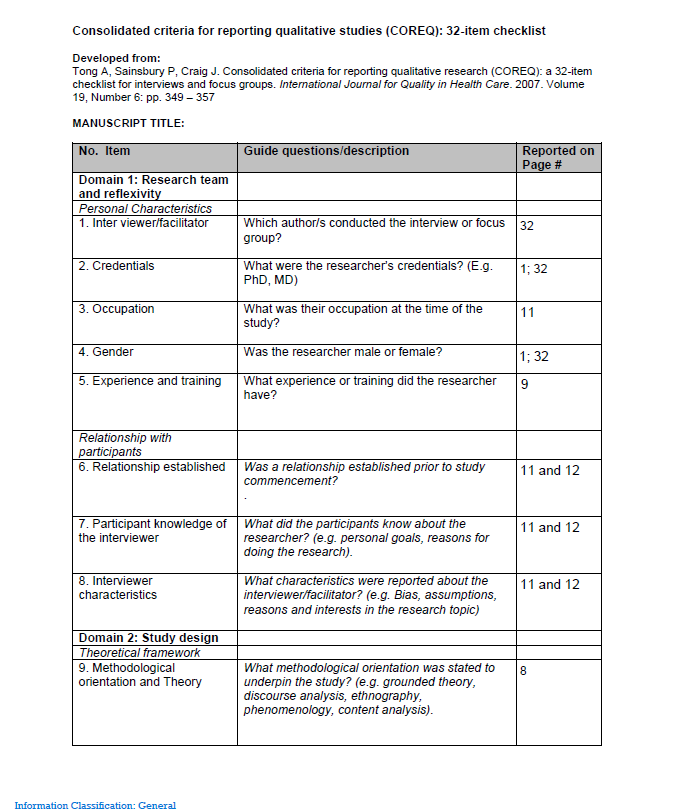
**

**
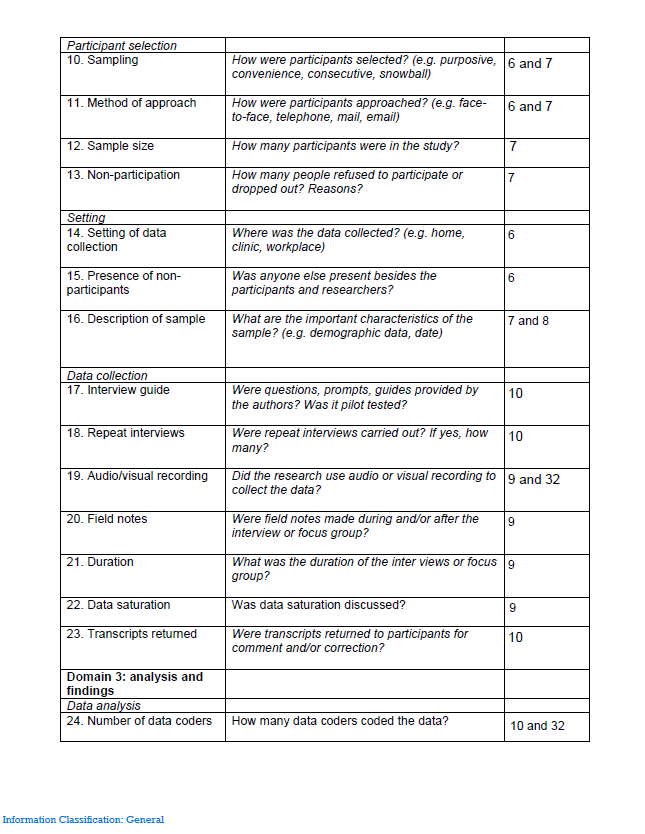
**

**
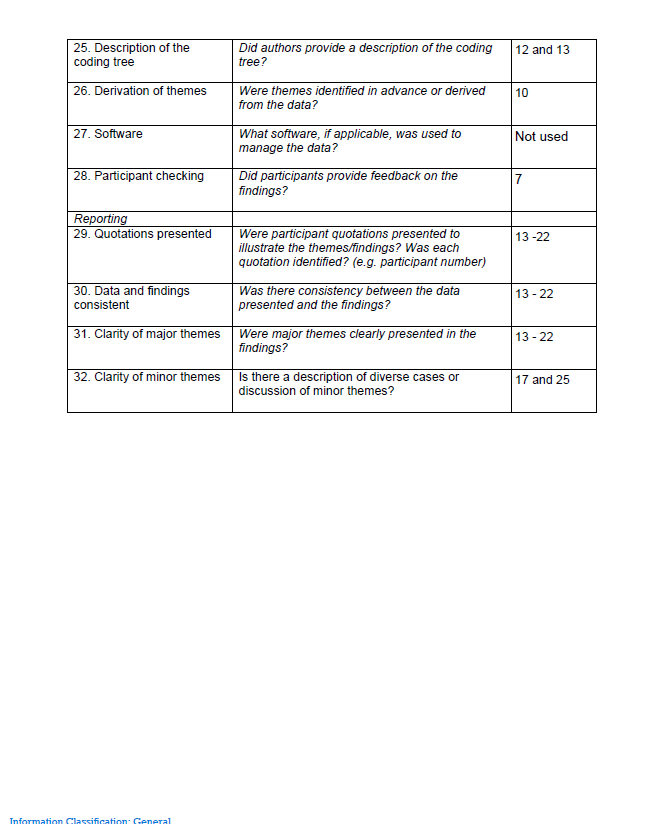
**

Supplement: Checklist 1 [file rehab-v12-e71789-s002.docx]
